# Mitochondrial dysfunction in neurodegenerative disorders: mechanisms and therapeutic advances

**DOI:** 10.1186/s43556-026-00480-x

**Published:** 2026-05-28

**Authors:** Yan Tong, Jing Na He, Linbin Zhou, Jiaxin Zhang, Bo Man Ho, Lin Du, Yolanda Wong Ying Yip, Hemlata Bisnauthsing, Poemen P. Chan, Clement C. Tham, Chi Pui Pang, Wai Kit Chu

**Affiliations:** 1 Department of Ophthalmology and Visual Sciences, The Chinese University of Hong Kong, Hong Kong Eye Hospital, 147 K Argyle Street, Kowloon, Hong Kong, China; 2 Department of Ophthalmology, Shenzhen People’s Hospital (The First Affiliated Hospital, Southern University of Science and Technology; The Second Clinical Medical College, Jinan University), Shenzhen, Guangdong China; 3 Lam Kin Chung. Jet King-Shing Ho Glaucoma Treatment and Research Centre, The Chinese University of Hong Kong, Hong Kong, China; 4 Joint Shantou International Eye Center of Shantou University and The Chinese University of Hong Kong, Shantou, 515041 China

**Keywords:** Mitochondrial dysfunction, Neurodegenerative diseases, Oxidative phosphorylation, Gene therapy, Mitochondrial dynamics, Clinical translation

## Abstract

Mitochondrial dysfunction is a core pathogenic mechanism underlying a broad spectrum of neurodegenerative disorders, from Alzheimer’s and Parkinson’s diseases to inherited optic neuropathies and mitochondrial ataxias. This review provides a comprehensive analysis of how defects in mitochondrial and nuclear DNA converge to disrupt oxidative phosphorylation, mitochondrial dynamics, calcium homeostasis, and quality control pathways, leading to energy depletion, oxidative stress, and neuronal degeneration across multiple disease contexts. Building on this mechanistic foundation, we examine how these shared pathogenic principles manifest distinctly in major neurodegenerative diseases, while also discussing representative mitochondrial optic neuropathies as tractable disease models that have yielded critical mechanistic and therapeutic insights. We further review recent advances in diagnostic technologies that enhance our ability to detect and stratify mitochondrial pathologies for therapeutic intervention. On the therapeutic front, we provide a comprehensive evaluation of the rapidly evolving landscape, analyzing strategies ranging from metabolic modulators and antioxidants to pioneering gene-targeted therapies, organelle replacement approaches, and emerging epitranscriptomic interventions. Finally, we identify persistent challenges in clinical translation and outline pivotal future directions essential for developing effective, mechanism-informed combination therapies against mitochondrial dysfunction in neurodegeneration.

## Introduction

Presenting in virtually all eukaryotes, mitochondria are involved in the regulation of multiple important pathways in cells, particularly in neurons [1]. As the primary source of adenosine triphosphate (ATP), generated via oxidative phosphorylation (OXPHOS), mitochondria are fundamental to meet the high bioenergetic demands of the nervous system. Consequently, dysfunction stemming from defects in mitochondrial (mtDNA) or nuclear DNA (nDNA)-encoded proteins constitutes a core pathogenic mechanism driving neuronal loss across neurodegenerative disorders [2]. This core mechanism reflects the neuron’s reliance on mitochondrial bioenergetics, calcium homeostasis, and dynamic quality control [3, 4].

Mitochondrial dysfunction has emerged as a unifying pathological hallmark across neurodegenerative diseases, from the aggregation-driven proteinopathies such as Alzheimer’s and Parkinson’s diseases to the motor neuron degeneration in amyotrophic lateral sclerosis (ALS), the polyglutamine toxicity in Huntington’s disease (HD), and the iron-sulfur cluster biogenesis defect in Friedreich’s ataxia (FRDA) [5]. In each context, bioenergetic failure, oxidative stress, disrupted mitochondrial dynamics, and defective quality control converge to amplify neuronal vulnerability and drive disease progression.

**Fig. 1:**
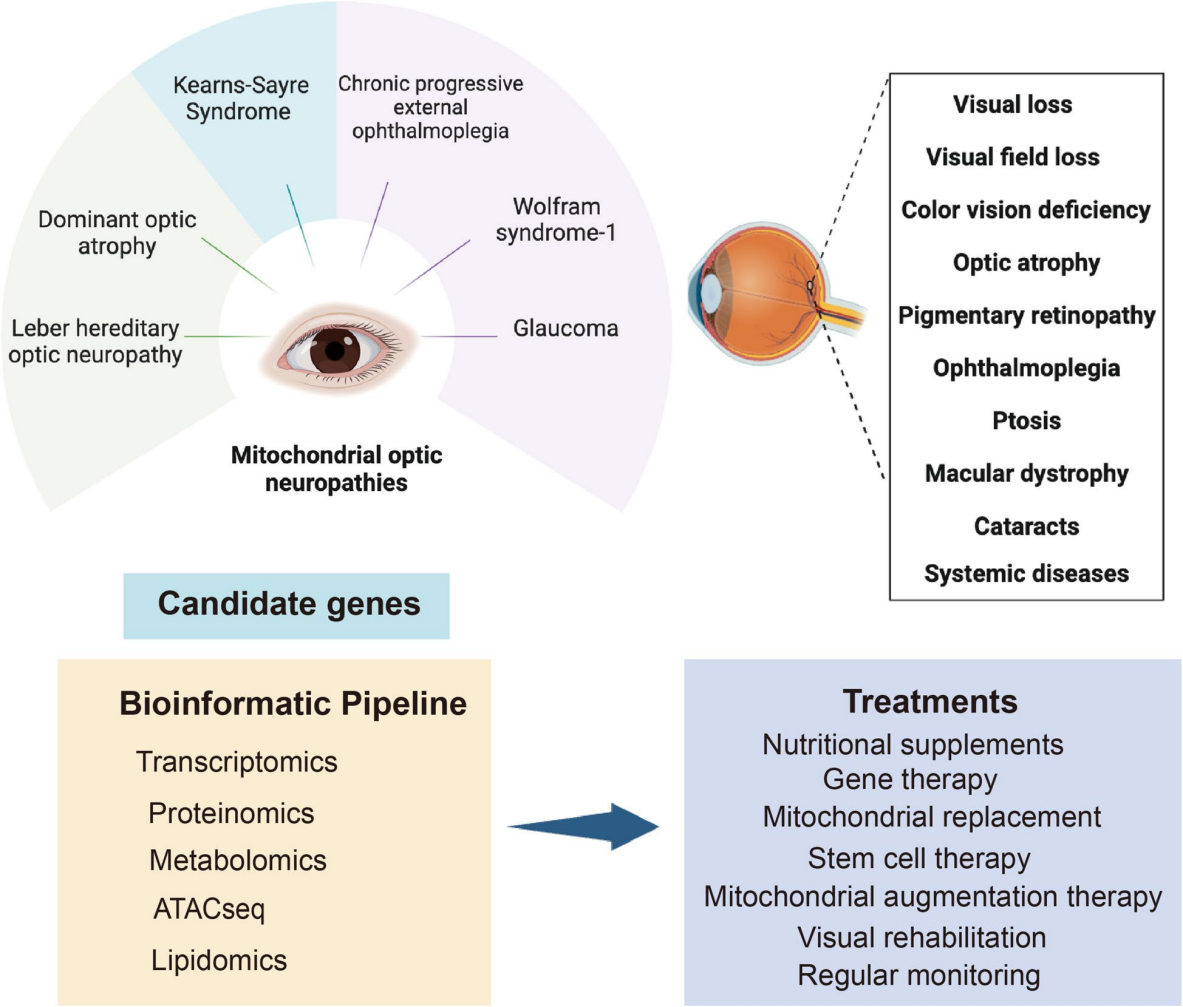
Schematic diagram on the mitochondrial optic neuropathies and associated therapeutic strategies. This figure offers a comprehensive overview of mitochondrial optic neuropathies, highlighting common visual disturbances. It also illustrates methods for detecting candidate genes and outlines various therapeutic approaches aimed at managing these conditions

Within this landscape, the visual system emerges as a uniquely powerful model for dissecting fundamental principles of mitochondrial neurodegeneration. In human eyes, the network of neurons and photoreceptors are responsible for processing electrical signals, which require significant amounts of energy, making these cells particularly vulnerable to mitochondrial defects. Hereditary optic neuropathies encompass a diverse range of disorders primarily characterized by progressive visual impairment (Fig. 1). The estimated global prevalence of the conditions is approximately 1 in 10,000 individuals [6]. The genetic etiology of these neuropathies is heterogeneous, arising from pathogenic variants in both mtDNA and nDNA genomes. The well-defined genetic etiologies, confined neuroanatomical involvement, and stereotypic clinical progression of these optic neuropathies make them invaluable for dissecting how specific mitochondrial defects lead to neuronal dysfunction, yielding insights that inform our understanding of more complex neurodegenerative conditions.

This review aims to construct a conceptual bridge from molecular mechanisms to clinical applications. We begin by systematically outlining the core mechanisms of mitochondrial dysfunction across multiple neurodegenerative disorders and summarizing recent advances in diagnostic and monitoring tools. We then examine mitochondrial pathology across a broad spectrum of neurodegenerative disorders, including major systemic diseases and representative mitochondrial optic neuropathies. Finally, we critically evaluate current therapeutic limitations and propose future directions for mitochondria-targeted interventions across the full breadth of neurodegeneration.

## Mechanistic aspects of mitochondrial dysfunction in neurodegeneration

### Dual-genomic control of mitochondrial bioenergetics

The pathogenesis of mitochondrial dysfunction is rooted in the failure of core physiological processes essential for neuronal survival. Foremost among these is OXPHOS, the cornerstone of mitochondrial bioenergetics that meets the extraordinary metabolic demands of neurons. This ATP-generating system is distinguished by its unique genomic architecture and serves as a critical regulatory node integrated with broader mitochondrial homeostatic networks.

Mitochondria bioenergetic competence is governed by a synergistic, bi-genomic system. They contain multiple copies of their own 16,569 bp circular mtDNA. mtDNA is located within the mitochondrial matrix, which is the internal compartment bounded by the mitochondrial inner membrane. Thirteen proteins encoded by the mtDNA are crucial subunit components of the OXPHOS enzymatic complexes. The translation of these mitochondrial proteins requires two mitochondrial specific ribosomal RNAs (rRNAs) and 22 transfer RNA (tRNA). Most mitochondrial genes are located on the H-strand, while only *MTND6* and 8 tRNA genes are found on the L-strand [7]. The majority of the mitochondrial proteome, encompassing the remaining 80 OXPHOS subunits, assembly factors, and apparatus for mtDNA replication, transcription, and repair, is encoded by the nuclear genome. Consequently, this dual-genomic control necessitates exquisite anterograde (nucleus-to-mitochondria) and retrograde (mitochondria-to-nucleus) signaling. Disruption of this coordinated cross-talk, through mutations in both genomes, constitutes the primary genetic etiology of disorders such as Leber hereditary optic neuropathy (LHON) and dominant optic atrophy (DOA) [8].

**Fig. 2:**
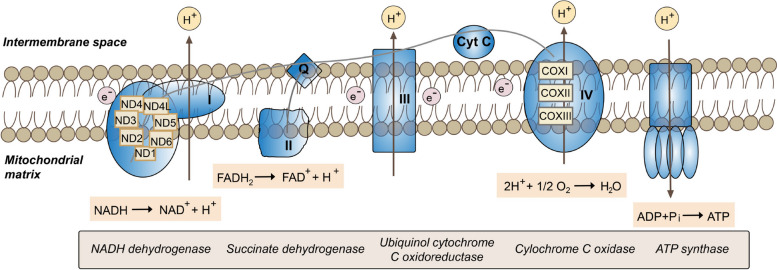
The mitochondrial respiratory chain and oxidative phosphorylation. The schematic illustrates the multi-subunit enzyme complexes (Complexes I–IV) embedded in the inner mitochondrial membrane that transfer electrons and pump protons to establish an electrochemical gradient. This proton-motive force is subsequently utilized by ATP synthase (Complex V) to drive cellular ATP production

The synthesis of ATP is executed by the OXPHOS, which is composed of four multi-subunit polypeptide complexes (complexes I-IV) that are embedded within the inner mitochondrial membrane along with the ATP synthase (complex V) (Fig. 2). Acetyl-CoA, an intermediate product of β-oxidation and glycolysis, is metabolized by the tricarboxylic acid cycle to generate flavin adenine dinucleotide hydrogen (FADH_2_) and nicotinamide adenine dinucleotide hydrogen (NADH). NADH and FADH_2_ then donate electrons to complex I and II, respectively, for re-oxidation. Electrons are then shuttled through the chain by the mobile carrier’s ubiquinone (CoQ10) and cytochrome c, which undergo successive redox reactions through Complex III and Complex IV, respectively. The resulting electrochemical gradient across the inner mitochondrial membrane is utilized by complex V (ATP synthase) to catalyze the conversion of adenosine diphosphate (ADP) and inorganic phosphate (Pi) to ATP.

### Disruption of mitochondrial homeostasis: from quality control to cell death

Mitochondrial dysfunction in neurodegeneration extends beyond isolated defects in OXPHOS. A growing number of evidence indicates that the pathology primarily arises from the disruption of integrated homeostatic systems, which subsequently couple bioenergetic failure to neuronal degeneration and death.

Mitochondrial homeostasis is maintained through quality control mechanisms, including mitophagy, fusion-fission dynamics, and biogenesis. Mitophagy, a selective form of autophagy responsible for the recycling and degradation of mitochondria, can be classified into three primary subtypes: PINK1-Parkin-dependent, receptor-mediated (involving BNIP3 and FUNDC1), and lipid-mediated pathways [9]. PINK1-Parkin-dependent mitophagy is initiated by mitochondrial membrane depolarization, whereas receptor-mediated mitophagy operates independently of ubiquitin and can be induced by hypoxia or developmental signals. Additionally, certain lipids such as cardiolipin and ceramide can directly regulate mitophagy by facilitating cargo recognition.

Mitochondrial dynamics (fission and fusion) also directly influences ATP synthesis efficiency [10]. Mitochondrial fission is initiated by pre-constriction via the endoplasmic reticulum and actin cytoskeleton, which creates a binding site for the key regulator DRP1. Recruited to the outer mitochondrial membrane (OMM) by adaptor proteins MFF and FIS1, DRP1 oligomerizes in a GTP‑dependent manner, ultimately leading to membrane scission. In contrast, mitochondrial fusion is mediated by GTP‑dependent dimerization of MFN1-MFN2 at the outer membrane and OPA1 at the inner membrane. Modulating mitochondrial dynamics thus represents a promising upstream strategy for fine‑tuning mitophagy [11].

Furthermore, the regulation of cytosolic calcium ion (Ca^2+^) is a core signaling function in mitochondria. Sustained Ca^2+^ overload can trigger the opening of the mitochondrial permeability transition pore (mPTP), leading to electron leakage and mitochondrial ROS (mtROS) elevation [12]. Excessive matrix Ca^2+^ can also stimulate inflammatory responses. During mPTP opening and oxidative stress, damage-associated molecular patterns [including cytochrome c, high-mobility group box 1 (HMGB1), and mtDNA] are released from the intermembrane space, which can drive innate immune responses. mtDNA can activate the cGAS-STING pathway, promoting NLRP3 inflammasome assembly and type I interferon production. Cytochrome c can initiate caspase-9-dependent apoptosis via apoptosome formation. HMGB1 can exacerbate inflammation by engaging toll-like receptors (TLRs) and the receptor for advanced glycation end products (RAGE) [13]. Consequently, even minor perturbations in the ATP/ADP ratio or the redox state can dictate cell fate, shifting metabolism toward adaptive signaling, metabolic reprogramming, or programmed cell death.

### Emerging paradigms: novel mechanisms and druggable targets

Beyond these established pathways, recent discoveries have unveiled novel mechanistic layers and therapeutic targets, expanding the intervention landscape for mitochondrial optic neuropathies. For instance, the paradigm of mitochondrial quality control has been revolutionized by the discovery of developmentally programmed purifying selection. The ubiquitin-specific peptidase 30 (USP30) was identified as a key brake on PINK1-Parkin-dependent mitophagy during the maternal-zygotic transition. Pharmacological inhibition of USP30 unleashes this latent mitophagic capacity, enabling the selective reduction of high-heteroplasmy mutant mtDNA loads in experimental models [14]. This strategy shifts the goal from merely supporting dysfunctional mitochondria to actively “cleansing” the mitochondrial pool, offering a preventive approach for mutation carriers.

Furthermore, studies have increasingly focused on the interface between mitochondrial dynamics and cellular architecture. The anchoring of mitochondria to the endoplasmic reticulum (ER) at mitochondrial ER contact sites (MERCs) is crucial for calcium signaling, lipid transfer, and fission initiation. Disruption of MERCs proteins, such as MFN2 can lead to aberrant calcium flux and fission, implicating this subcellular microdomain in disease pathogenesis [15]. Another layer of precision in quality control is revealed by mitochondrial-derived vesicles (MDVs), which mediate the targeted delivery of damaged components to lysosomes or peroxisomes, operating independently from mitophagy [16]. Dysregulation in MDV biogenesis thus leads to the accumulation of specific toxic cargo.

Collectively, these advances highlight that mitochondrial dysfunction extends beyond bioenergetics to encompass failures in spatial organization, piecemeal quality control, and inter-organelle communication, each representing a new frontier for therapeutic intervention.

## Advances in diagnostic technologies for mitochondrial dysfunction in neurodegeneration

The broad genetic and clinical heterogeneity inherent to mitochondrial disorders renders definitive diagnosis a persistent challenge, necessitating a convergent strategy that spans fluid-based biomarkers, high-resolution DNA sequencing, functional neuroimaging, and histopathological interrogation. When mitochondrial disease is suspected, initial biochemical profiling of blood, urine, and cerebrospinal fluid constitutes the first diagnostic tier. Elevated lactate, arising from compensatory glycolytic flux when oxidative phosphorylation is compromised, is frequently observed but diagnostically imperfect; the lactate-to pyruvate ratio, which mirrors the cytosolic NAD^+^/NADH redox equilibrium, provides a substantially greater discriminatory power [17]. Among circulating protein biomarkers, growth differentiation factor 15 (GDF15) and fibroblast growth factor 21 (FGF21), both upregulated by the mitochondrial integrated stress response, have demonstrated a robust diagnostic performance for mitochondrial myopathies, with additional promise as longitudinal indicators of treatment efficacy. Plasma cell-free mtDNA and extracellular vesicles carrying mitochondrial cargo are also under active evaluation as accessible molecular indicators of mitochondrial stress [18, 19].

Several conventional diagnostic techniques based on clinical features, biochemical screening, and skeletal muscle biopsies have been utilized for the diagnosis of neurodegenerative disorders. However, these approaches often yield a high number of false negative or false positive results, as well as being costly, thereby limiting their usefulness in diagnosing and monitoring diseases. Recent advancements in genetic sequencing methods have improved the effectiveness in detecting mtDNA mutations and other mitochondrial abnormalities. The conventional Sanger DNA sequencing method has emerged as a predominant technique for detecting DNA mutations [20]. Over the last few decades, the Sanger method has undergone significant advancements and automation, facilitating large-scale DNA sequencing initiatives like Human Genome Project. Sanger sequencing can also be utilized for analyzing mtDNA point mutations and deletion across multiple genes, including those encoded by mtDNA itself [21]. This method provides a reliable approach for detecting specific mutations that may contribute to various mitochondrial disorders.

Next-generation sequencing technologies have emerged as a significant advancement to provide a rapid identification approach for the detection of mtDNA disorders. It has significantly increased the volume of sequence data, enabling the sequencing of entire coding sequences or even the whole genome at a lower cost [22]. Various next-generation sequencing methods, such as targeted-exome sequencing, whole-exome sequencing, whole-genome sequencing, RNAseq, and whole mtDNA sequencing, have effectively alleviated concerns related to genetic variants and diseases. Subsequently, the duplex sequencing has been discovered to be over 10,000-fold more accurate than the conventional next generation sequencing. Duplex sequencing examines both stands of DNA and identifies mutations only if they occur as complementary substitutions in both strands of a single DNA molecule. This advancement enables precise mutation analysis for the complete set of mtDNA in cells [23].

Another novel approach called MitoRS has been developed for the detection of mtDNA variants. This technique uses rolling circle amplification to amplify the whole mitochondrial genome in a single reaction, eliminating the need for primers or temperature regulation, thereby achieving high sensitivity and accuracy [24]. Furthermore, Lareau et al. recently developed a single-cell multi-omic method named mtscATAC-seq (mitochondrial single-cell assay for transposase-accessible chromatin with sequencing). This technology allows the characterization of accessible chromatin while simultaneously performing high-throughput genotyping of mtDNA. It offers a versatile means of identifying the genetic connections among multiple cells within human tissues, exploring fundamental aspects of mitochondrial genetics, and facilitating multi-omic discoveries [25].

Beyond genomic profiling, functional neuroimaging enables non-invasive, spatially resolved assessment of mitochondrial bioenergetic status. Fluorodeoxyglucose positron emission tomography (FDG-PET) detects early reductions in cerebral glucose metabolism that reflect underlying mitochondrial bioenergetic deficits, serving as preclinical biomarker in Alzheimer’s disease [26]. Magnetic resonance spectroscopy quantifies reduced N-acetylaspartate levels as potential indicators of impaired oxidative phosphorylation, with demonstrated utility in Huntington’s disease [27]. In optic neuropathies, optical coherence tomography enables micrometer-scale quantification of retinal nerve fiber attrition as a structural correlation of ongoing neurodegeneration. When molecular testing is non-diagnostic, skeletal muscle biopsy remains the reference standard [28]. Sequential cytochrome c oxidase/succinate dehydrogenase histochemistry reveals mosaic patterns of segmental respiratory chain deficiency, while high-resolution respirometry quantifies oxygen consumption attributable to individual complexes [29]. The integration of these multi-modal diagnostic platforms with genomic data promises to enable earlier detection, more precise patient stratification, and objective therapeutic monitoring in mitochondrial neurodegeneration.

## Mitochondrial dysfunction and therapeutic strategies across neurodegenerative diseases

The following sections systematically examine the manifestations of mitochondrial dysfunction across major neurodegenerative disorders, ranging from widespread central nervous system diseases to instructive optic neuropathies. While these conditions differ markedly in their clinical presentation and neuropathological topography, they converge on shared hallmarks of mitochondrial pathophysiology. For each disorder, we integrate the molecular basis of disease-specific mitochondrial vulnerability with an appraisal of the therapeutic landscape, encompassing approved agents, advanced clinical candidates, and emerging preclinical strategies, thereby providing a unified mechanistic–therapeutic framework across the spectrum of mitochondrial neurodegeneration.

### Alzheimer’s disease (AD)

AD, one of the most common neurodegenerative disorders, is clinically characterized by progressive cognitive decline and pathologically defined by extracellular senile plaques composed of amyloid-β (Aβ) peptides and intracellular neurofibrillary tangles of hyperphosphorylated tau [30]. Although the amyloid and tau hypotheses have historically dominated the field, accumulating evidence positions mitochondrial dysfunction as a central, early event in AD pathogenesis. The "mitochondrial cascade hypothesis" posits that an individual’s baseline mitochondrial function dictates AD risk; consequently, the age-related decline in mitochondrial efficiency acts as a primary upstream trigger for amyloidogenesis and tau pathology, rather than a mere downstream byproduct [31, 32].

A defining macroscopic feature of AD is the early and progressive reduction in cerebral glucose metabolism, which is detectable via fluorodeoxyglucose positron emission tomography years prior to clinical symptom onset [33]. Underpinning this metabolic deficit at the molecular level is the profound suppression of key mitochondrial enzymes, including pyruvate dehydrogenase, α-ketoglutarate dehydrogenase, and cytochrome c oxidase (complex IV) [34]. As Aβ accumulate within mitochondria (imported via the translocase of the outer membrane complex), they directly interact with mitochondrial membranes, physically disrupting complex IV activity, impairing the electron transport chain, and exacerbating oxidative stress [35]. Concurrently, tau pathology compounds these metabolic deficits by disrupting microtubule-associated trafficking, thereby impeding the axonal transport of mitochondria and precipitating synaptic energy starvation [36]. Reinforcing this microtubule–mitochondria axis, fibroblast growth factor 13 (FGF13) has been identified as a stabilizer of mitochondrial function via direct interaction with β-tubulin isotype IIA (TUBB2A). In AD animal models, promoter hypermethylation silences FGF13, leading to microtubule destabilization and mitochondrial membrane depolarization, defects reversed by FGF13 restoration but abolished by TUBB2A knockdown [37].

**Fig. 3:**
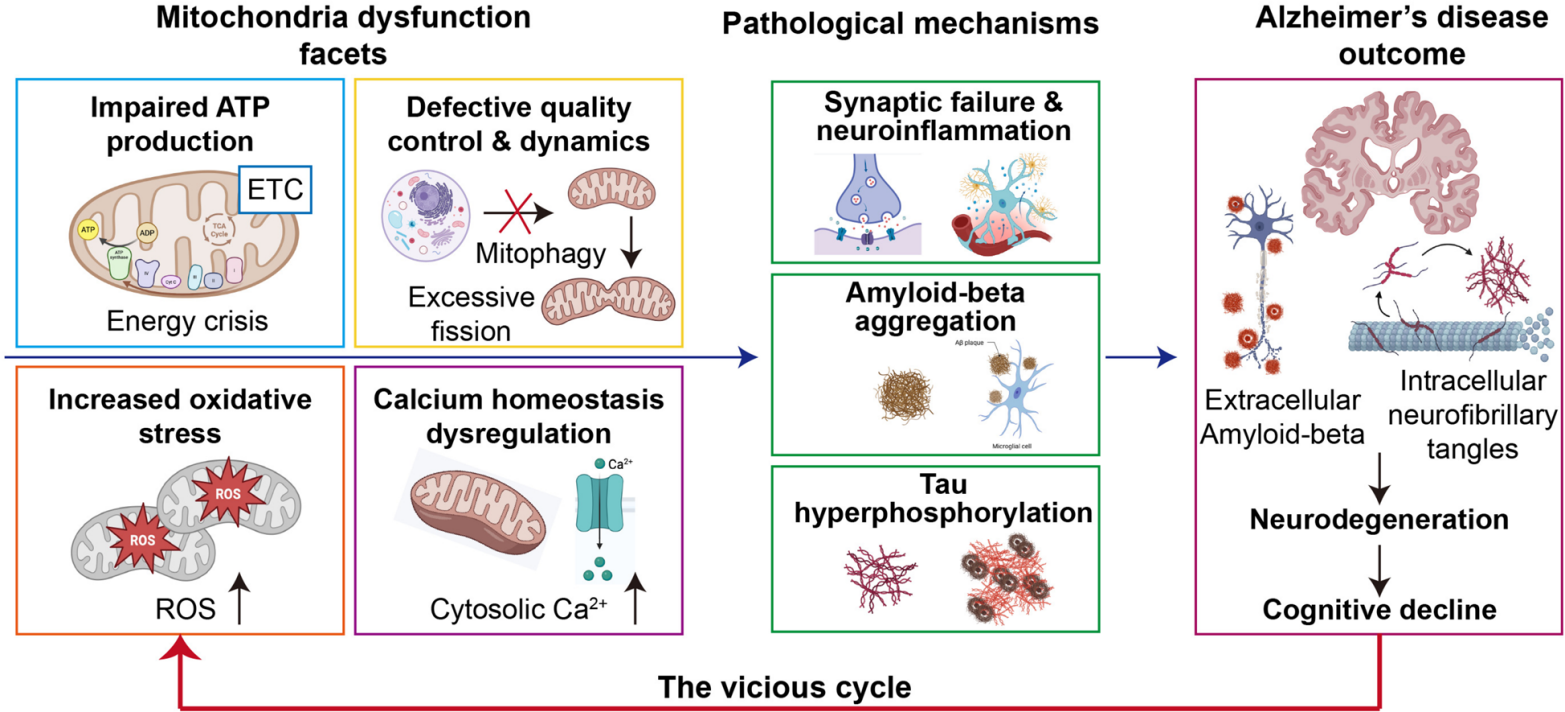
The vicious cycle of mitochondrial dysfunction and AD pathogenesis. The multifaceted nature of mitochondrial dysfunction is characterized by impaired ATP production leading to an energy crisis, defective mitochondrial quality control, increased oxidative stress driven by ROS, and dysregulation of cytosolic calcium homeostasis. These mitochondrial defects act as upstream drivers for key AD pathological mechanisms, including synaptic failure, neuroinflammation, amyloid-beta aggregation, and tau hyperphosphorylation. The progression of these mechanisms results in the hallmark AD outcomes: the accumulation of extracellular Aβ plaques and intracellular neurofibrillary tangles, which collectively drive neurodegeneration and subsequent cognitive decline. The red feedback loop highlights how established AD pathology further exacerbates mitochondrial dysfunction, perpetuating disease progression. ATP, adenosine triphosphate; ETC, electron transport chain; ROS, reactive oxygen species; Aβ, amyloid-beta; TCA, tricarboxylic acid cycle

As illustrated in Fig. 3, the oxidative stress and metabolic imbalance induced by Aβ oligomers profoundly disrupt the delicate equilibrium between mitochondrial fusion and fission. The downregulation of key fusion mediators (e.g., OPA1, MFN1, MFN2) paired with the upregulation of fission proteins (for example DRP1, FIS1, MFF) drives excessive mitochondrial fragmentation [38, 39]. Crucially, the clearance of these fragmented mitochondria is severely compromised. Multiple mitophagy pathways including the PINK1-Parkin axis, receptor-mediated pathways (BNIP3 and FUNDC1), and AMBRA1-initiated mechanisms are defective in AD [40]. Reinforcing this defect, the RNA demethylase ALKBH3 (AlkB homolog 3), upregulated in AD patient brains, removes N1-methyladenosine (m1A) from PINK1 mRNA, destabilizing this key mitophagy regulator [41]. Furthermore, the initiation of macroautophagy is suppressed through the inhibition of the Beclin1-VPS15-VPS34 complex [42]. This dual failure of excessive mitochondrial fragmentation coupled with deficient clearance results in the toxic accumulation of damaged, ROS-generating mitochondria. Consequently, this accumulation feeds a vicious cycle that exacerbates oxidative stress, accelerates tau hyperphosphorylation, and ultimately drives synaptic failure and neuronal apoptosis.

In addition, the disruption of calcium homeostasis at the ER-mitochondria interface is increasingly recognized as a critical pathogenic driver. Presenilin mutations, which account for the majority of familial AD cases, enhance ER calcium release via inositol trisphosphate receptors, leading to mitochondrial calcium overload [43]. This overload triggers the opening of the mPTP, the release of cytochrome c, and the activation of apoptotic cascades. Furthermore, emerging evidence indicates that Aβ oligomers physically disrupt mitochondria-associated ER membranes (MAMs). This disruption alters localized lipid metabolism and cholesterol trafficking, detrimentally modifying membrane composition and further crippling mitochondrial integrity [44]. Corroborating this pathogenic axis, recent studies using cortical neurons derived from patient induced pluripotent stem cells (iPSCs) have demonstrated that presenilin-1 mutations (F105C and A246E) induce pronounced mitochondrial defects, which are remarkably rescued by amyloid precursor protein (APP) knockout, identifying APP as a critical bridging mediator of familial AD-associated mitochondrial dysfunction [45].

Contemporary therapeutic strategies targeting mitochondrial dysfunction in AD operate across multiple mechanistic levels (Table 1). Mitochondria-targeted antioxidants, such as SS-31 (elamipretide), stabilize inner membrane cardiolipin to improve respiratory chain efficiency and mitigate initial oxidative triggers. Concurrently, DRP1 inhibitors (for example Mdivi-1) aim to rebalance mitochondrial dynamics by attenuating the pathological fragmentation. Mitophagy enhancers, such as Urolithin A, seek to restore the clearance of damaged organelles; notably, Urolithin A has ameliorated mitochondrial and cognitive deficits in preclinical AD animal models and is currently under clinical evaluation in elderly cohorts [56]. Additionally, ongoing clinical trials are assessing metabolic modulators including oxaloacetate and ketogenic interventions to provide alternative bioenergetic substrates that bypass the impaired neuronal glucose metabolism [57]. Crucially, recent multi-omics investigations highlight that mitochondrial dysfunction-related biomarkers in the cerebrospinal fluid, such as altered acylcarnitine profiles and diminished cytochrome c oxidase activity, hold promise as early diagnostic indicators of treatment response [58]. Ultimately, the therapeutic objective extends beyond rescuing individual mitochondria to restoring the integrated metabolic and signaling networks whose collapse drives neurodegeneration.

**Table 1:** Summary of key mitochondria-targeting compounds that can potentially treat Alzheimer’s disease

Candidate drugs	Mechanisms of action	Primary targets/pathways
Mdivi-1 [46]	Allosteric inhibitor of DRP1 GTPase; suppresses pathological fission	Mitochondrial fission inhibitor
CP2 [47]	Potent DRP1 inhibitor; suppresses pathological fragmentation	Mitochondrial fission inhibitor
Urolithin A [48]	Induces mitophagy, enhances mitochondrial function and turnover	Mitophagy inducer
Spermidine [49]	Natural polyamine; induces autophagy via epigenetic mechanisms	Autophagy/mitophagy inducer
Rapamycin [50]	Inhibits mTORC1, potently induces autophagy and mitophagy	mTOR inhibitor/autophagy inducer
MitoQ [51]	Conjugated to TPP⁺, accumulates in mitochondria, scavenges mtROS	Mitochondria-targeted antioxidant
Mito VitE [51]	Vitamin E conjugated to TPP⁺; protects mitochondrial membranes from lipid peroxidation	Mitochondria-targeted antioxidant
SS-31 (Elamipretide) [52]	Stabilizes ETC super-complexes, reduces ROS, inhibits mPTP	Mitochondrial inner membrane stabilizer
NDI1 [53]	Yeast NADH dehydrogenase; re-establishes electron flow when Complex I is impaired	Alternative dehydrogenase
Metformin [54]	Activates AMPK, improves insulin sensitivity; may enhance mitophagy and biogenesis	AMPK activator/metabolic modulator
Resveratrol [55]	Activates SIRT1, upregulates PGC-1α; enhances mitochondrial biogenesis & oxidative defense	Sirtuin activator/biogenesis

*DRP1* Dynamin-related protein 1, *mTORC1* mechanistic Target of Rapamycin Complex 1, *TPP⁺* Triphenyl phosphonium cation, *mPTP* Mitochondrial permeability transition pore, *NDI1* NADH dehydrogenase (internal) 1, *AMPK* AMP-activated protein kinase, *SIRT1* Sirtuin 1, *PGC-1α* Peroxisome proliferator-activated receptor-gamma coactivator 1-alpha

### Amyotrophic lateral sclerosis (ALS)

ALS is clinically defined by the progressive degeneration of both upper and lower motor neurons, culminating in fatal respiratory failure [59]. ALS exemplifies a profound axonal bioenergetic catastrophe, extending the principle of mitochondrial vulnerability to extreme demands of the motor system.

The key pathology lies in the failure of mitochondrial logistics and local bioenergetic maintenance within motor neuron axons. Core ALS-linked proteins, such as mutant SOD1, mislocalized TDP-43, and dipeptide repeat proteins produced by the C9orf72 hexanucleotide expansion, directly impair the axonal transport machinery [60–62]. This disruption impedes the anterograde delivery of functional mitochondria to distant synaptic terminals and neuromuscular junctions, leading to chronic energetic depletion at these critical sites. Meanwhile, these pathological proteins induce intrinsic mitochondrial dysfunction at the soma and axons, characterized by calcium buffering defects, increased ROS, and decreased ATP production [63, 64].

Mechanistically, mutant SOD1 accumulates on the OMM and within the intermembrane space. Here, it interacts with the voltage-dependent anion channel 1 (VDAC1), crippling the electron transport chain and driving the vacuolar degeneration of mitochondria, a pathological hallmark in SOD1 transgenic models [65]. Furthermore, mutant SOD1 disrupts ER-mitochondria calcium signaling by binding to Bcl-2 at MAMs, thereby promoting calcium overload and triggering apoptotic cascades [66]. Recent evidence also highlights the propensity of toxic SOD1 oligomers to physically associate with and mechanically disrupt the integrity of the OMM [67].

This mitochondrial targeting is a convergent feature across multiple ALS-linked mutations. TDP-43, pathologically mislocalized in over 90% of all ALS cases, translocates into mitochondria and binds mitochondrial mRNA transcripts encoding complex I subunits, thereby impairing their translation and disassembling the respiratory complex. Moreover, TDP-43 sequesters Parkin mRNA in the cytosol, potently inhibiting the PINK1-Parkin mitophagy pathway and arresting the clearance of damaged organelles [68]. Similarly, the C9orf72 repeat expansion generates poly-GR and poly-PR dipeptide repeats that preferentially accumulate within mitochondria, inhibit ATP synthase (complex V) activity, and dissipate the mitochondrial membrane potential [69]. Reinforcing this genetic convergence, FUS (fused in sarcoma), another ALS-linked RNA-binding protein, associates with mitochondrial ATP synthase and profoundly distorts cristae morphology upon mis-localization [70].

Critically, this neuronal energy crisis is markedly exacerbated by a parallel collapse of glial metabolic support. Astrocytes, which normally provide lactate and other metabolic substrates to motor neurons via the astrocyte-neuron lactate shuttle, develop their own mitochondrial dysfunction in ALS [71]. This impairs their ability to fuel neurons, particularly under stress. Furthermore, dysfunctional microglia and astrocytes adopt a pro-inflammatory phenotype, releasing cytokines and additional ROS that further damage neuronal mitochondrial integrity and function [72]. Thus, ALS pathology evolves from a cell-autonomous transport defect to a non-cell-autonomous metabolic network failure.

Therapeutic strategies for ALS therefore aim to address both the “logistical” and “energetic” dimensions of the crisis. Current efforts focus on enhancing axonal mitochondrial transport (for example using HDAC6 inhibitors to promote microtubule acetylation), boosting mitochondrial biogenesis and antioxidant defense via PGC-1α-NRF2 pathway activators, and exploring ways to restore glial metabolic support [73, 74]. Riluzole, the first approved ALS disease-modifying therapy, operates in part by attenuating glutamate excitotoxicity, thereby mitigating downstream mitochondrial calcium overload. Edaravone, a subsequently approved free radical scavenger, shields mitochondrial function from oxidative damage, demonstrating modest deceleration of functional decline in selective patient cohorts [75].

Emerging paradigms are shifting toward precision genetic medicines. Antisense oligonucleotide (ASO) therapies targeting mutant SOD1 mRNA (such as tofersen, FDA-approved in 2023) and C9orf72 repeat expansions aim to indirectly rescue mitochondrial function by reducing the upstream burden of toxic protein species [76]. The approval of tofersen represents a watershed moment, validating genetically targeted therapies that address the root causes rather than merely the downstream consequences of mitochondrial damage [77]. Additionally, advanced gene-silencing platforms are emerging as powerful tools for dissecting and mitigating mitochondrial toxicity in ALS models. A recently developed embedded CRISPR interference (emCRISPRi) system, which integrates transcriptional repression domains into catalytically inactive Cas9, has demonstrated robust attenuation of TDP-43-induced neurotoxicity in a Drosophila ALS model [78]. In parallel, metabolic interventions, including high-calorie diets and medium-chain triglyceride supplementation, are undergoing clinical evaluation to counteract the hypercatabolic state characteristic of ALS [79], a systemic reflection of the severe energetic crisis driven by motor neuron mitochondrial failure. Finally, experimental approaches such as mitochondrial transplantation, involving the direct transfer of healthy mitochondria to diseased cells, offer a novel, albeit nascent, therapeutic avenue in preclinical ALS models.

### Parkinson’s disease

Parkinson’s disease is a complex neurodegenerative disorder defined by progressive motor symptoms (including bradykinesia, rigidity, and tremor), and a spectrum of non-motor symptoms, which significantly impact patient’s quality of life [80]. Pathologically, Parkinson’s disease is characterized by the selective vulnerability and progressive loss of dopaminergic neurons in the substantia nigra pars compacta, accompanied by the accumulation of intraneuronal Lewy bodies rich in aggregated α-synuclein. Historically, the profound connection between mitochondria and Parkinson’s disease was serendipitously uncovered when 1-methyl-4-phenyl-1,2,3,6-tetrahydropyridine (MPTP), an environmental toxin and potent mitochondrial complex I inhibitor, was found to induce rapid-onset parkinsonism in humans [81].

**Fig. 4:**
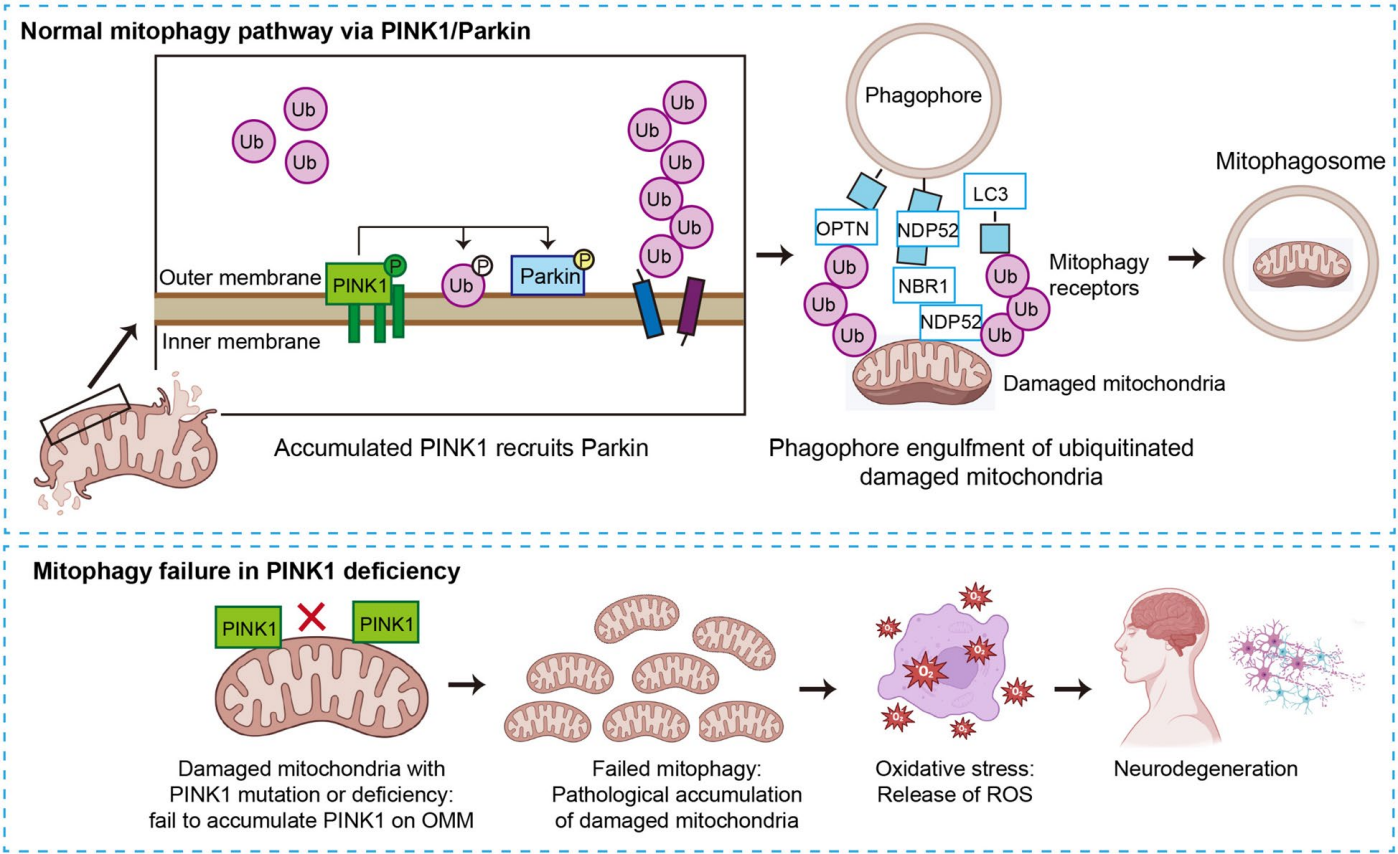
PINK1/Parkin-mediated mitophagy and its failure in Parkinson’s disease. Normal mitophagy: In healthy mitochondria, PINK1 is rapidly degraded. Upon mitochondrial damage, PINK1 stabilizes on OMM and recruits Parkin. Parkin then mediates the ubiquitination of mitochondrial proteins, triggering the engulfment of the damaged mitochondrion by a phagophore to form a mitophagosome for clearance. In PINK1 deficiency or mutation, mutant PINK1 fails to accumulate on OMM of damaged mitochondria. This prevents Parkin recruitment and downstream ubiquitination, completely blocking the mitophagy pathway. Consequently, damaged mitochondria pathologically accumulate in neuronal cells. LC3, microtubule-associated protein 1A/1B-light chain 3; OMM, outer mitochondrial membrane; PINK1, PTEN-induced kinase 1; p-Ub, phosphorylated ubiquitin; ROS, reactive oxygen species

Familial Parkinson’s disease genetics has provided compelling evidence for the primacy of mitochondrial dysfunction. Mutations in PINK1 and Parkin, two of the most common causes of autosomal recessive Parkinson’s disease, directly impair the PINK1-Parkin mitophagy pathway [82]. Under physiological conditions, the kinase PINK1 is stabilized on the outer membrane of depolarized mitochondria, subsequently recruiting and activating the E3 ubiquitin ligase Parkin to flag the damaged organelle for autophagic clearance. Loss-of-function mutations in either gene short-circuit this critical PINK1-Parkin mitophagy pathway, leading to the toxic accumulation of damaged mitochondria, elevated ROS, and ultimate dopaminergic demise (Fig. 4). Furthermore, mutations in *LRRK2*, the most common cause of autosomal dominant Parkinson’s disease, impair mitochondrial dynamics and promote DRP1-mediated mitochondrial fission [83], while mutations in *DJ-1* compromise mitochondrial antioxidant defense. More recently, loss-of-function mutations in phospholipase A2 group VI (PLA2G6), another genetic cause of autosomal recessive Parkinson’s disease, have been shown to destabilize the IP3R1–GRP75–VDAC1 tethering complex at MAMs, reducing ER–mitochondria contacts and impairing calcium transfer in patient iPSC-derived dopaminergic neurons, which could be rescued by an artificial MAM linker [84]. Importantly, even in sporadic Parkinson’s disease, which accounts for approximately 90% of cases, complex I deficiency in the substantia nigra is a consistent finding.

The crucial mitochondrial pathology in Parkinson’s disease is a self-reinforcing cycle of bioenergetic failure, defective quality control, and oxidative stress. The hallmark is the specific deficiency of mitochondrial complex I in the substantia nigra, leading to impaired ATP production and increased electron leakage [85, 86]. This bioenergetic crisis is compounded by dysregulated mitochondrial dynamics, including excessive DRP1-mediated fission and impaired MFN1-MFN2-OPA1-mediated fusion, which fragments the mitochondrial network [87]. The resultant accumulation of dysfunctional mitochondria drives elevated ROS and disrupts calcium buffering, further exacerbating neuronal stress and death. Adding an epitranscriptomic layer to this cycle, m6A RNA hypomethylation, driven by reduced METTL3 expression in substantia nigra dopaminergic neurons, impairs mitochondrial biogenesis through downregulation of the mtDNA transcription factor Tfam, while the resulting mitochondrial dysfunction reciprocally suppresses m6A deposition, creating a self-perpetuating pathogenic loop [88].

Recent insights reveal that the progression of Parkinson’s disease is accelerated by the dysfunction of aging glial cells [89]. Critically, a compensatory mtDNA maintenance mechanism present in healthy aging brains is impaired, leading to progressive mtDNA depletion and respiratory chain failure [90]. In parallel, aging microglia undergo a pro‑inflammatory phenotypic shift, driven in part by the down‑regulation of TREM2 and impaired CX3CL1-CX3CR1 signaling [91]. This compromises their ability to clear α‑synuclein aggregates and neurotoxic debris, while promoting the release of inflammatory cytokines. Simultaneously, aging astrocytes exhibit intrinsic mitochondrial dysfunction that can activate the cGAS–STING–YY1 pathway, further amplifying neuroinflammation [92]. Together, these alterations establish a vicious paracrine loop: mitochondrial‑stressed neurons release damage signals that activate glial cells, which in turn release cytotoxic factors that further undermine neuronal and mitochondrial integrity [93].

Current therapeutic development proceeds along several rational axes, including strategies to enhance mitophagy such as USP30 inhibitors (discussed in Section “Emerging paradigms: novel mechanisms and druggable targets”) and ursodeoxycholic acid, which aim to artificially boost the clearance of damaged mitochondria in patients with residual PINK1-Parkin function [94]. Concurrently, bioenergetic and dynamic rescue interventions seek to restore metabolic homeostasis using complex I chaperones, DRP1 inhibitors (for example Mdivi-1), and precision metabolic rescue like NDI1 gene therapy designed to entirely bypass the defective host complex I [95]. While traditional antioxidants like Coenzyme Q10 have yielded mixed clinical results, emphasizing the need for biomarker-driven patient stratification. Emerging neuroinflammatory modulators (for example TREM2 agonists or cGAS-STING inhibitors) are actively being deployed to quell the secondary glial-driven toxicity triggered by mitochondrial DNA release [92, 96]. In parallel, a novel "organelle therapy" strategy employing erythrocyte membrane-encapsulated mitochondrial capsules has demonstrated rescue of dopaminergic neuron loss, motor recovery, and restoration of mitochondrial function in mouse models, offering a translatable platform for direct mitochondrial replacement [97]. Finally, epidemiological studies consistently linking pesticide exposure (specifically direct complex I inhibitors like rotenone and paraquat) to elevated Parkinson’s disease risk not only validate the mitochondrial hypothesis from an environmental perspective but also underscore that mitochondrial protection strategies harbor immense value for both acute therapeutic intervention and long-term disease prevention.

### Huntington’s Disease (HD)

Huntington’s disease (HD) is an autosomal dominant neurodegenerative disorder caused by a CAG trinucleotide repeat expansion in the *HTT* gene, encoding a polyglutamine-expanded mutant huntingtin (mHTT) protein [98]. Clinically characterized by progressive chorea, cognitive decline, and psychiatric disturbances, HD exhibits a striking neuropathological vulnerability: the preferential degeneration of medium spiny neurons (MSNs) in the striatum. Unlike disorders driven primarily by protein aggregation at the organelle level, HD is fundamentally a disease of profound systemic metabolic reprogramming initiated by early nuclear transcriptional dysregulation.

At the heart of this metabolic collapse is the direct interference of mHTT with the function of PGC-1α, the master transcriptional coactivator of mitochondrial biogenesis and oxidative metabolism [99]. By sequestering the *CREB/TAF4* complex at the *PGC-1α* promoter, mHTT profoundly suppresses its transcription, precipitating a global downregulation of nuclear-encoded mitochondrial genes [100]. This selectively disturbs the electron transport chain (particularly complexes II and III) and forces a deleterious shift toward inefficient glycolytic metabolism [101]. Such a bioenergetic deficit proves especially catastrophic for striatal MSNs given their exceptionally high basal energy demands, a finding corroborated by reduced complex activities in postmortem tissues and pathologically elevated basal ganglia lactate levels observed via magnetic resonance spectroscopy. Reinforcing this paradigm, recent metabolomic profiling highlights widespread perturbations in the TCA cycle, amino acid metabolism, and lipid oxidation pathways. Notably, recent evidence has identified the one-carbon (1C) metabolic pathway as a critical node of dysregulation in HD. Specifically, the mitochondrial enzyme SHMT2 is significantly downregulated in HD animal models, leading to the accumulation of homocysteine [102]. This metabolic shift does not merely impair bioenergetics but acts as an epigenetic rheostat; elevated homocysteine interacts with AARS1 to suppress histone lactylation, thereby driving a pathological transcriptional program that exacerbates mHTT aggregation and striatal degeneration.

Beyond transcriptional repression, mHTT actively dismantles organelle homeostasis through aberrant calcium handling and impaired mitochondrial dynamics [103]. At MAMs, mHTT sensitizes the inositol 1,4,5-trisphosphate receptor (IP3R), exacerbating ER calcium release, while simultaneously impairing mitochondrial calcium uptake via the mitochondrial calcium uniporter complex [104]. The ensuing cytosolic calcium overload dramatically heightens MSN susceptibility to glutamate-mediated excitotoxicity and lowers the threshold for mPTP opening.

HD is also associated with excessive mitochondrial fragmentation, driven by mHTT-induced upregulation of DRP1 and its enhanced recruitment to mitochondria via direct protein–protein interaction [87]. Simultaneously, mHTT disrupts axonal mitochondrial transport by interfering with the huntingtin-HAP1-dynein/kinesin motor complex, leading to the depletion of mitochondria at synaptic terminals [105]. This transport defect results in synaptic energy failure that precedes overt neurodegeneration, mirroring the early synaptic dysfunction observed clinically. Importantly, wild-type huntingtin normally plays an essential role in facilitating mitochondrial transport, and the loss of this normal function, compounded by the toxic gain-of-function from the polyglutamine expansion, creates a dual hit on mitochondrial logistics within vulnerable striatal neurons.

Finally, these profound bioenergetic and dynamic deficits are exacerbated by elevated oxidative stress, originating from both compromised respiratory chain function and calcium-induced ROS overproduction. Consequently, biomarkers of oxidative damage, including 8-hydroxy-2’-deoxyguanosine (8-OHdG) and malondialdehyde, are significantly elevated in both the plasma and brain tissue of HD patients [106]. Critically, this oxidative burden directly targets mtDNA, driving the accumulation of deletions and point mutations that further impair respiratory chain subunit assembly, thereby entrenching a vicious degenerative feedback loop. Furthermore, this metabolic failure is linked to a collapse in proteostasis, characterized by the accumulation of mHTT aggregates that further deplete cellular resources. Recent mechanistic insights suggest that deubiquitinating enzymes, specifically UCHL3, act as gatekeepers of this process; elevated UCHL3 activity in HD models appears to impede autophagosome-lysosome fusion, thereby stabilizing toxic polyQ fragments [107]. Conversely, inhibiting UCHL3 promotes aggregate clearance and induces a STAT3-dependent stress response, suggesting that restoring the balance between protein degradation and mitochondrial health is essential for neuronal survival.

Emerging therapeutic strategies for HD increasingly target the transcriptional-metabolic axis to rescue cellular bioenergetics and mitochondrial dynamics. Pharmacological interventions aimed at restoring mitochondrial biogenesis include PGC-1α activators and bezafibrate, a pan-PPAR agonist, while inhibitors of mitochondrial fission are being explored to counteract pathological network fragmentation [108]. Complementary to these approaches, cysteamine has demonstrated clinical promise by concurrently enhancing brain-derived neurotrophic factor secretion and bolstering mitochondrial complex II activity [109]. Metabolic vulnerabilities are also being addressed through the provision of alternative energy substrates, for instance, ketogenic diets supply non-glucose fuels, and triheptanoin, an anaplerotic medium-chain triglyceride, is being investigated for its capacity to replenish TCA cycle intermediates [110]. Further bioenergetic strategies include the use of succinate prodrugs to bypass complex II deficiencies and mitochondria-targeted antioxidants, such as MitoQ, to mitigate localized oxidative stress. Upstream of these pathways, gene-silencing modalities utilizing ASOs and RNA interference to suppress mHTT expression offer the potential to indirectly rescue mitochondrial function by lifting the transcriptional repression of key metabolic genes [111]. However, recent clinical setbacks with the safety concerns in a phase III study of ASO tominersen underscore the profound complexities of successfully translating these mHTT-lowering therapies.

### Multiple Sclerosis (MS)

Multiple sclerosis (MS) is a chronic inflammatory demyelinating and neurodegenerative disease of the central nervous system. While classically considered as an autoimmune disorder targeting myelin, progressive mitochondrial dysfunction is increasingly recognized as a central driver of the irreversible neurodegeneration that characterizes the progressive phases of the disease [112]. Activated immune cells within MS lesions release reactive oxygen and nitrogen species, particularly nitric oxide, which directly inhibits mitochondrial complex IV and induces irreversible damage to mtDNA. This is supported by histopathological studies showing marked reduction in complex I and complex IV activity in demyelinated axons within chronic active MS lesions [113]. Furthermore, TNF-α and other pro-inflammatory cytokines impair mitochondrial membrane potential and promote mPTP opening in both neurons and oligodendrocytes. Notably, recent evidence highlights the critical role of granzyme B-expressing CD8 + T cells in driving MS progression [114]. Mechanistically, these lesion-infiltrating cells can directly transfer granzyme B to trigger mitochondrial-mediated apoptosis in target oligodendrocytes and neurons.

Oligodendrocytes are exceptionally dependent on oxidative phosphorylation for the production of myelin lipids and membranes required for repair. Their mitochondria are highly susceptible to inflammation-induced damages, including nitrosative stress and calcium overload [115]. This leads to oligodendrocyte metabolic exhaustion, failure of remyelination, and subsequent axonal degeneration. Demyelinated axons must redistribute sodium channels along their entire length to maintain conduction, dramatically increasing their energy demands. The Na +/K + -ATPase required to restore ionic gradients after action potential propagation along bare axons consumes vastly more ATP than in myelinated fibers. Without adequate mitochondrial support, these axons undergo progressive degeneration, a process termed “virtual hypoxia” [116]. This concept is supported by evidence of tissue hypoxia-like changes in acute MS lesions, including upregulation of HIF-1α and its downstream targets.

A striking finding in MS is the accumulation of clonally expanded mtDNA deletions in neurons of the cortex and deep gray matter, similar to patterns seen in aging but markedly accelerated [117]. These deletions impair respiratory chain function, particularly complex IV, creating a mosaic of respiratory-deficient neurons that are preferentially vulnerable to further injury. Importantly, the burden of mtDNA deletions correlates with neuronal density loss in MS cortex, suggesting a direct pathogenic role. This convergence of inflammation-driven and age-related mitochondrial damage explains the transition from relapsing–remitting to progressive MS, where neurodegeneration becomes increasingly independent of acute inflammatory activity. Additionally, mitochondrial dysfunction in MS extends to the grey matter, where cortical demyelination is associated with extensive mitochondrial injury in neurons [117], astrocytes, and oligodendrocyte precursor cells, limiting their capacity for differentiation and remyelination.

MS therapeutic strategies focus on combining immunomodulation with neuroprotection. Agents that dampen inflammatory damage to mitochondria include mitochondria-targeted antioxidants such as MitoQ, which has shown neuroprotective effects in experimental autoimmune encephalomyelitis animal models by reducing oxidative damage and preserving axonal integrity. Biotin (MD1003), which acts as a cofactor for mitochondrial carboxylases and is thought to enhance myelin repair and energy production, has been evaluated in progressive MS clinic trials with preliminary positive signals, though subsequent larger trials yielded less consistent results. Simvastatin has demonstrated neuroprotective effects in secondary progressive MS, potentially through enhancement of mitochondrial function, reduction of oxidative stress, and anti-inflammatory actions [118]. Clemastine, an antihistamine repurposed for its remyelinating properties, promotes oligodendrocyte survival and differentiation by targeting Gsta4 to suppress the mitochondria-associated Casp8-Bid apoptotic axis [119]. Furthermore, mesenchymal stem cell therapy is being investigated for its dual immunomodulatory and neuroprotective properties, with evidence suggesting that transplanted cells can transfer functional mitochondria to injured neurons and oligodendrocytes via tunneling nanotubes [120].

### Friedreich’s Ataxia

Friedreich’s ataxia (FRDA) is the most common inherited ataxia, with a prevalence of approximately 1 case per 50,000 people [121]. It is an autosomal recessive disorder caused predominantly by homozygous GAA trinucleotide repeat expansions in intron 1 of the *FXN* gene, which encodes frataxin, a small mitochondrial protein essential for iron-sulfur (Fe-S) cluster biogenesis [122]. Unlike the proteinopathies discussed above, FRDA represents a primary mitochondrial biogenesis disorder where a single upstream deficiency in Fe-S cluster assembly cascades into multi-system mitochondrial failure, making it a paradigmatic disease for understanding mitochondrial iron metabolism in neurodegeneration.

**Fig. 5:**
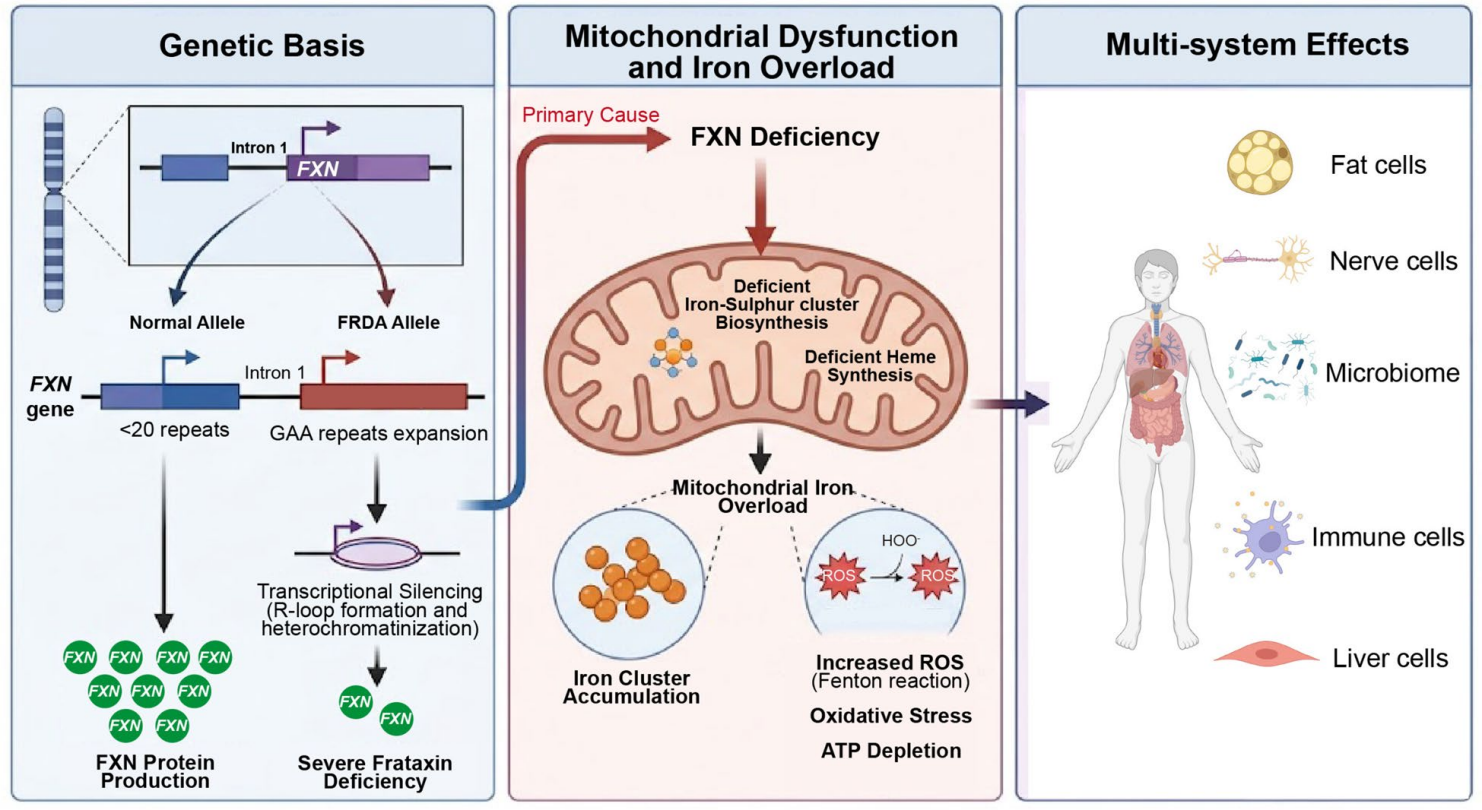
Molecular pathogenesis and multi-systemic impact of Friedreich’s Ataxia (FRDA). The genetic hallmark of FRDA involves a GAA trinucleotide repeat expansion within Intron 1 of the *FXN* gene. Unlike the normal allele, the expanded FRDA allele undergoes transcriptional silencing through R-loop formation and heterochromatinization, resulting in severe FXN protein deficiency. As a primary cause of cellular pathology, frataxin deficiency severely disrupts mitochondrial function, specifically impairing iron-sulfur cluster biosynthesis and heme synthesis. This disruption leads to profound mitochondrial iron overload, characterized by iron cluster accumulation and increased generation of ROS via the Fenton reaction. The resulting oxidative stress and ATP depletion severely compromise cellular viability. Ultimately, these subcellular mitochondrial defects manifest as multi-systemic clinical effects, negatively impacting adipose tissue, the nervous system, the microbiome, immune cells, and hepatic function. FXN, frataxin; FRDA, Friedreich’s Ataxia; ROS, reactive oxygen species; ATP, adenosine triphosphate

The GAA repeat expansion induces heterochromatin formation and transcriptional silencing of *FXN*, reducing frataxin protein levels to 5%–30% compared to healthy people. Frataxin functions as an iron chaperone in the mitochondrial matrix, facilitating Fe-S cluster assembly on the scaffold protein ISCU2 within the iron-sulfur cluster machinery [123]. Fe-S clusters are essential prosthetic groups for multiple enzymes in the electron transport chain (complexes I, II, and III) and the TCA cycle (aconitase), as well as for DNA repair enzymes [124]. Consequently, frataxin deficiency leads to impaired activity of Fe-S cluster-containing enzymes, resulting in reduced OXPHOS efficiency, decreased ATP production, and compromised aconitase activity (Fig. 5). This bioenergetic deficit particularly affects tissues with high metabolic demands, including dorsal root ganglia neurons, cardiomyocytes, and cerebellar dentate nucleus neurons.

A defining pathological feature of FRDA is the paradoxical accumulation of iron within mitochondria despite systemic iron dysregulation. Impaired Fe-S cluster synthesis leads to iron retention in the mitochondrial matrix, where it catalyzes Fenton reactions generating highly toxic hydroxyl radicals. This iron-mediated oxidative stress damages mitochondrial DNA, proteins, and lipid membranes, creating a feed-forward cycle of mitochondrial deterioration. Recent studies have demonstrated that mitochondrial iron overload in FRDA also activates ferroptosis, a regulated cell death pathway driven by iron-dependent lipid peroxidation, providing a mechanistic link between iron dyshomeostasis and neuronal loss [125]. Furthermore, frataxin deficiency disrupts NRF2-mediated antioxidant responses, reducing the expression of key detoxifying enzymes such as superoxide dismutase 2, glutathione peroxidase, and catalase, thereby diminishing the cell’s capacity to counteract the elevated oxidative burden [126].

FRDA presents in childhood or adolescence with progressive gait and limb ataxia, dysarthria, loss of deep tendon reflexes, and proprioceptive sensory loss. Hypertrophic cardiomyopathy develops in the majority of patients and represents the leading cause of death, typically occurring in the third or fourth decade of life [121]. The cardiac pathology reflects mitochondrial dysfunction in cardiomyocytes, with iron deposits, respiratory chain deficiency, and fibrosis observed histopathologically. Diabetes mellitus develops in approximately 30% of patients, attributed to mitochondrial dysfunction in pancreatic β cells, paralleling the diabetic phenotype seen in Wolfram syndrome. Optic neuropathy and sensorineural hearing loss occur in a subset of patients, providing a further link to the mitochondrial optic neuropathies discussed below.

Therapeutic approaches for FRDA target multiple nodes of the pathogenic cascade. Omaveloxolone (Skyclarys), an NRF2 activator, became the first FDA-approved therapy for FRDA in 2023 [127], based on clinical trial data demonstrating improvement in neurological function as measured by the modified Friedreich’s Ataxia Rating Scale (mFARS). Omaveloxolone acts by activating the NRF2 pathway to enhance mitochondrial antioxidant defenses and reduce oxidative stress. Deferiprone, an iron chelator capable of crossing the blood–brain barrier and redistributing mitochondrial iron [128], has shown preliminary benefits in cardiac parameters in clinical trials, though neurological outcomes remain under investigation. Gene therapy approaches aim to restore frataxin expression, with AAV-mediated FXN gene delivery demonstrating efficacy in cardiac and neuronal tissues in mouse models [129]. Emerging strategies include epigenetic derepression of the silenced *FXN* gene using histone deacetylase inhibitors and small molecules that counteract GAA repeat-mediated heterochromatin formation, which could potentially restore endogenous frataxin expression without the need for exogenous gene delivery [130]. Additionally, recent preclinical work has explored etravirine, an antiretroviral drug repurposed for its ability to upregulate frataxin protein levels via a post-translational mechanism [131], highlighting the expanding pharmacological landscape for treating this disorder.

### The visual system as an instructive model: mitochondrial optic neuropathies

The visual system serves as a powerful and instructive model for understanding mitochondrial biology in neurodegeneration. Ophthalmic disorders, particularly mitochondrial optic neuropathies, offer a uniquely tractable paradigm. Their well-defined genetic etiology, confined neuroanatomical involvement, and stereotypic clinical progression allow a clear dissection of how specific mitochondrial defects lead to neuronal dysfunction and loss. The following subsections highlight representative conditions whose mechanistic and therapeutic insights inform the broader field.

#### Leber hereditary optic neuropathy (LHON)

**Fig. 6:**
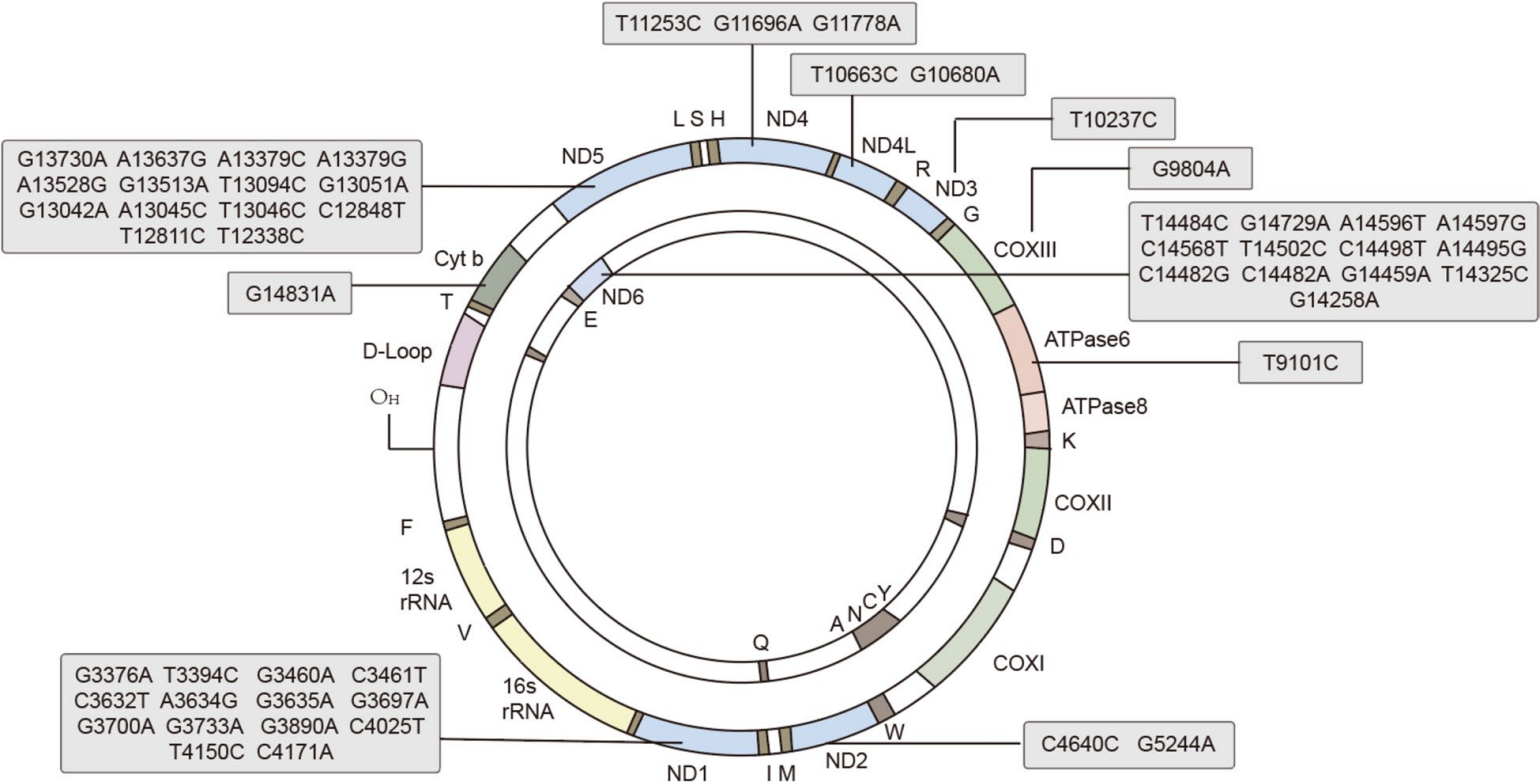
The schematic diagram of the human mitochondrial genome and the variants’ associated with LHON. Protein coding, tRNA, and rRNA genes are shown on the heavy (outer) and light (inner) strands. Genes encoding subunits of complex I (ND1 to ND6) are represented in blue. The subunits of the ATP synthase (ATPase 6 and 8) are displayed in red, while the cytochrome c oxidase (COXI to COXIII) is displayed in green. The displacement loop (D-loop), also known as the non-coding control region, contains crucial sequences for initiating both mtDNA transcription and replication, including the proposed origin of heavy-strand replication (OH). The two rRNAs (12 s rRNA and 16 s rRNA) which are essential for protein synthesis within the mitochondria, are displayed in yellow; 22 tRNA are indicated by brown lines and denoted by their cognate amino acid letter code

LHON is caused by primary mutations in mtDNA that affect the subunits of complex I in the mitochondrial respiratory chain. The most common mtDNA mutations found in over 90% of LHON patients are m.3460G > A, m.11778G > A, and m.14484 T > C mutations, affecting subunits 1, 4, and 6 of the mitochondrial NADH dehydrogenase respectively [132]. These mutations lead to a lower efficiency of ATP synthesis and an excessive production of ROS. Around 95% of LHON cases are associated with one of these three major mutations (Fig. 6). LHON has a sex bias towards males, exhibit incomplete penetrance, and can be modified by environmental factors such as alcohol consumption, nutritional deficiencies, and tobacco smoking [133]. LHON typically presents as sequential or bilateral subacute painless optic neuropathies that result in subacute irreversible vision loss, with optic disc hyperemia and telangiectatic vessels acutely, progressing to pallor. Swept-source optical coherence tomography imaging shows marked thinning of the choroidal layer, correlating with the retinal ganglion cell-inner plexiform layer and the retinal nerve fiber layer (RNFL) thickness [134].

Intensive studies have been conducted to investigate therapeutic molecules that counterbalance the accumulation of ROS and improve ATP production, using distinct combinations of vitamins and supplements such as carnitine, alpha-lipoic, vitamin C, and creatine. Among these, Idebenone, a short-chain and synthetic analogue of ubiquinone, has emerged as the most established pharmacotherapy for LHON. It is activated to its oxidized form in the cytoplasm by NAD(P)H:quinone oxidoreductase (NQO1), allowing shuttle of electrons directly to complex III, bypassing the malfunctioning complex I. Increased ATP production and reduced ROS levels have been observed in LHON fibroblast cell lines treated with Idebenone [135]. A multi-center, randomized clinical trial in 85 LHON patients experiencing vision loss within five years provided evidence that individuals with more recent onset of vision loss and discordant visual acuities are more likely to benefit from Idebenone treatment [136]. Following this study, Carelli et al. reported that earlier visual improvement was related to longer duration of Idebenone treatment [137]. The European Union authorized the use of Idebenone in LHON patients in 2015. And the consensus conference in 2017 recommended that Idebenone treatment should be initiated as soon as possible in patients with disease onset of less than one year [138]. Additional compounds including alpha-tocotrienol quinone (EPI-734), cyclosporine A, and elamipretide (MTP-131) [139, 140] have been developed to alleviate the neurotoxic stress posed on retinal ganglion cells (RGCs) by ROS.

Gene therapy for LHON uses intravitreal injection of AAV vectors to deliver wild-type mitochondrial genes allotopically. Since various DNA molecules cannot cross the mitochondrial membranes without assistance, the transfected DNA is commonly delivered to the nuclear genome to express genes for allotopic expression. A wild-type version of the mutated mitochondrial gene is expressed in the cytoplasm and the resulting protein is then transported into mitochondria to restore its physiologic functions [141]. Protective effects of allotopic ND4 gene therapy, including preventing the loss of RGCs, restoring ATP synthesis and preserving vision, have been demonstrated in the experimental model of LHON [142]. Several clinical trials (including phase I, II, and III) for LHON gene therapy have been conducted (Table 2). Guy et al. evaluated the safety of escalated doses of scAAV2-P1ND4v2 in a phase I clinical trial, showing improved average visual acuity with no serious adverse events [143]. A phase I/II trial delivered rAAV2/2-ND4 (GS010 LUMEVOQ) via single unilateral intravitreal injection into the worse-seeing eye of 15 LHON patients, observing functional visual benefit and RGCs preservation [144]. The phase III RESCUE (vision loss up to 6 months) and REVERSE (vision loss > 6 months to 1 year) evaluated GS010 in 76 patients with m.1178G > A mutation [145, 146]. While the primary endpoint of a 15 “Early Treatment Diabetic Retinopathy Study (ETDRS)” letter improvement in best-corrected visual acuity (BCVA) was not met in RESCUE, a gradual visual recovery was observed. The REVERSE trial showed similar trends in both treated and sham-treated eyes, suggesting unexpected bilateral improvements. The long-term RESTORE follow-up (61 patients, 3 years post-treatment) demonstrated sustained improvements in BCVA and vision-related quality of life lasting up to 4.3 years [147].

**Table 2:** Completed and ongoing clinical trials (2010 to present) for the treatment of LHON

*Clinical Trial Number*	*Study details*	*Type*	*Intervention strategies; time since visual loss onset*	*Age at onset*	*Mutations*	*Measurements*
**Drug treatment**						
NCT04561466	Study of efficacy of Befizal for the treatment of LHON	Interventional, phase 2/3 clinical trial	Oral Befizal (600 mg/day) for one year; LHON has occurred for less than 5 years	Subjects aged ≥ 18 years old	G3460A mutation; G11778A mutation	BCVA, retinal nerve fiber layer, and visual field up to 12 months
NCT02774005	Efficacy and safety of Idebenone in patients with LHON	Interventional, phase 4, open-label clinical trial	Oral Idebenone; onset of symptoms ≤ 5 years of Baseline	Subjects aged ≥ 12 years old	G11778A mutations; G3460A mutations or T14484C mutations	Safety and BCVA up at 12 months
NCT04381091	Expanded access program for Idebenone in patients with LHON who completed the LEROS study	Interventional, non-randomized clinical trail	Oral Idebenone (900 mg/day)	No limitation	Not reported	Safety and BCVA after treatment
NCT02693119	Safety, tolerability, and efficacy of Elamipretide topical ophthalmic solution for treatment of LHON	Phase 2, prospective, randomized, double-masked, vehicle controlled, single-center clinical trial	Elamipretide (MTP-131) topical ophthalmic solution twice daily; Loss of vision in both eyes of ≥ 1 year and ≤ 10 years	Subjects aged between 18 to 50 years old	G11778A mutation	AEs and BCVA up to 160 weeks
NCT02300753	Emergency administration of EPI-743 to a single patient with LHON	Interventional open-label study	Oral EPI-743 treatment	Subjects aged between 8 to 65 years old	Not reported	Safety and BCVA after treatment
NCT00528151	Efficacy and safety study of the curcumin treatment of LHON	Randomized, Phase 3, double-blind, placebo-controlled Trial	Oral curcumin (250 mg twice a day)	Subjects aged ≥ 8 years old	G11778A mutation	Visual outcome at 1 year after treatment
**Gene therapy**						
NCT05820152	Gene therapy clinical trial for the treatment of LHON associated with ND1 mutations	Phase 1/2, multi-regional, single-arm, open-label, dose-finding clinical trial	Single unilateral IVT injection of NFS-02 (rAAV-ND1); reduced VA lasted for > 6 months and < 10 years	Subjects aged ≥ 18 years old and ≤ 75 years old	G3460A mutation	Incidence of AEs, SAEs, DLT within 52 weeks; BCVA, contrast sensitivity, and visual evoked potential up to 65 months
NCT04912843	Gene therapy clinical trial for the treatment of LHON associated with ND4 mutations	Phase 1/2/3, multi-center, two-part clinical trial	Single unilateral IVT injection of NR082 (rAAV-ND4); reduced VA lasted for > 6 months and < 10 years	subjects aged ≥ 18 years old and ≤ 75 years old	G11778A mutation	Incidence rates of AEs, SAEs and DLT up to 52 weeks; BCVA, immunogenicity, and vector shedding/biodistribution up to 52 weeks
NCT03672968	Safety of GS010 in a single subject affected with LHON	Interventional, open-label study	Single bilateral IVT injection of GS010 (rAAV-ND4 treated one patient diagnosed with LHON	Not reported	G11778A mutation	Safety and BCVA after treatment
NCT02652767 (RESCUE)	Efficacy study of GS010 for the treatment of vision loss up to 6 months from onset in LHON due to the ND4 mutation	Randomized, double-masked, sham-controlled clinical trial	Single unilateral IVT injection of GS010 (rAAV2/2-ND4); vision loss is present for six months or less	Subjects aged ≥ 15 years old	G11778A mutation	Safety and BCVA at baseline and week 48
NCT02652780 (REVERSE)	Efficacy study of GS010 for treatment of vision loss from 7 months to 1 year from onset in LHON due to the ND4 mutation	Randomized, phase 3, double-masked, sham-controlled clinical trial	IVT injection of GS010 (rAAV2/2-ND4); vision loss is present for more than six months and up to one year	Subjects aged ≥ 15 years old	G11778A mutation	Safety and BCVA at baseline and week 48
NCT03406104 (RESTORE)	Long-term follow-up of ND4 LHON subjects treated with GS010 ocular gene therapy in the RESCUE or REVERSE Phase III clinical trials	Interventional, phase 3, open-label study	Single unilateral IVT injection of GS010 (rAAV-ND4)	Subjects aged ≥ 15 years	G11778A mutation	AEs and SAEs up to 5 years post-treatment; BCVA, visual field, spectral domain OCT parameters up to 5 years
NCT03428178	Efficacy study of gene therapy for the treatment of LHON	Interventional, open-label study	Single unilateral IVT injection of NR082 (rAAV2-ND4); acute LHON onset within three months	Subjects aged between 8 to 60 years old	G11778A mutation	BCVA, visual field, OCT, electroretinograms up to 12 months
NCT03293524	Efficacy and safety of GS010 for the treatment of LHON for up to one year	Phase 3, global, multi-center randomized, double-masked clinical trial	Single unilateral IVT injection of GS010 (rAAV2-ND4); vision loss duration is present up to one year	Subjects aged ≥ 15 years	G11778A mutation	BCVA at 1.5-year post baseline treatment; spectral-domain OCT parameters at 1.5 year and 2-year post baseline treatment
NCT03153293	A Single intravitreal injection of rAAV2-ND4 for the treatment of LHON	Interventional, phase 2/3, open-label, single-arm, multi-center clinical trial	Single unilateral IVT injection of NR082 (rAAV2-ND4)	Subjects aged between 10 to 60 years old	G11778A mutation	BCVA and visual field changes at baseline and 12 months
NCT02161380	Safety study of an adeno-associated virus vector for gene therapy of LHON	Interventional, phase 2, open-label, single-arm study	IVT injection of scAAV2-P1ND4v2	Subjects aged ≥ 15 years	G11778A mutation	AEs, SAEs and BCVA up to 3 years
NCT02064569	Safety evaluation of gene therapy (GS010) in LHON	Interventional, phase 1/2, open-label, single-arm study	IVT injection of GS0101 (rAAV-ND4)	Subjects aged ≥ 18 years	G11778A mutation	AEs, SAEs, and visual outcomes up to 48 weeks

*IVT* intravitreal, *VA* visual acuity, *AEs* adverse events, *SAEs* serious adverse events, *DLT* dose-limiting toxicity, *BCVA* best corrected visual acuity, *OCT* optical coherence tomography

Resources: https://www.clinicaltrials.gov

While both small molecule therapy and gene therapy represent significant advances, they differ substantially in their clinical profiles. Small molecule therapy offers non-invasive oral administration, broader patient eligibility, and manageable cost, but its efficacy is often partial and may require continuous, long-term administration. In contrast, gene therapy aims for a one-time, definitive intervention addressing the genetic root cause, with potential for sustained neuroprotection, though the clinical trials highlight challenges including the need for cautious evaluation given spontaneous recovery in LHON and relatively small sample sizes limiting generalizability.

The utilization of reprogrammed pluripotent stem cells derived from primary LHON patients’ cells provides a valuable tool for understanding and treating this disease [148]. CRISPR-Cas9 correction of the *YARS2* mutation has shown rescue of deficiencies in RGC-like cells differentiated from patient iPSCs [149]. Furthermore, the cultivation of retinal organoids from human pluripotent stem cells has facilitated the exploration of complex pathogenic mechanisms, enabling multiomic analyses at the single-cell level [150].

#### Dominant optic atrophy (DOA)

DOA, an inherited mitochondrial disease, is characterized by the progressive, bilateral, and predominant symmetric degeneration of RGCs and their descending axons. It follows an autosomal dominant inheritance pattern with prevalence ranging from 1 in 12,000 to 1 in 50,000 [151]. Although the onset of symptoms can vary significantly, more than 80% of patients exhibit some levels of optic nerve pallor during a fundus examination before the age of ten [152]. Patients experience gradual, painless decline in central visual acuity with color vision deficits, while peripheral vision is relatively spared [153]. A characteristic “wedge-shaped” temporal pallor in the optic nerve, reflecting early involvement of the papillomacular bundle, is a common diagnostic feature. Up to 20% of cases present as “DOA-plus” syndromes including ataxia, myopathy, hearing loss, and ophthalmoplegia [154].

Mutations in the *OPA1* gene can account for 65%–90% of DOA cases [155]. The *OPA1* gene is located on chromosome 3 (3q28-2940) and codes for the protein dynamin-related GTPase, which functions at the inner mitochondria membrane [156]. *OPA1* is associated with the inner mitochondrial membrane fusion, OXPHOS, calcium homeostasis, the stability of mtDNA, and the regulation of cytochrome c [157]. Over 400 distinct *OPA1* variants have been reported, spanning missense, frameshift, nonsense, splice-site, and structural variants [158]. The predominant disease mechanism is haploinsufficiency, resulting from variants that lead to premature termination codons and reduced protein levels. Marcela Votruba et al. demonstrated that a 50% reduction in *Opa1* transcript and protein correlates with marked synaptic loss in RGCs, despite the absence of soma loss [159]. They also investigated dendritic morphology in *Opa1*^±^ mutant mice, revealing age-related dendritic pruning in on-center RGCs without losing the cell bodies, illustrating that dendritic alterations occur early, potentially preceding clinical visual loss [160]. In contrast, specific missense mutations within the GTPase domain are strongly associated with more severe syndromic forms (DOA-plus) [161].

DOA also exhibits considerable genetic heterogeneity, with mutations in several other nuclear genes implicated in both isolated and syndromic forms [162] (Table 3). Intriguingly, specific *AFG3L2* missense variants clustering in the ATPase domain are linked to DOA, while variants in other protein regions cause spinocerebellar ataxia type 28, demonstrating a striking domain-specific disease association [168]. Genes like *SSBP1* (involved in mtDNA replication) and *NR2F1* (a transcriptional regulator) have been firmly established [175]. Nearly all DOA-associated genes converge on critical mitochondrial processes including fusion, fission, protein quality control and ribosome assembly, mtDNA maintenance, and cellular signaling. The clinical expression exhibits a broad spectrum, ranging from isolated optic neuropathy to complex neurodegenerative syndromes. This complex genotype–phenotype landscape underscores that DOA is not a single disorder but a continuum of diseases sharing a common pathway of optic nerve degeneration.

**Table 3:** Genetic factors underlying dominant optic atrophy: identified loci, gene functions, and associated clinical manifestations

*Genes*	*OMIM*	*Locus*	*Gene functions*	*Clinical manifestations*	*References*
*OPA1*	605290	3q28-q29	Mitochondrial biogenesis; mitochondrial fusion; and stabilization of mitochondrial membrane integrity	DOA	Alexander et al. [163]; Delettre C. et al. [164]
*OPA3*	606580	19q13.2-q13.3	Mitochondrial oxidative phosphorylation; mitochondrial network maintenance	DOA; cataract	Reynier et al. [165]
*MFN2*	608507	1p36.22	Mitochondrial fusion; mitophagy; mitochondrial motility, lipid transfer	Hereditary motor and sensory neuropathy type VI; Charcot-Marie-Tooth Disease Subtype 2A	Zuchner et al. [166, 167]
*SPG7*	602783	16q24.3	Mitochondrial quality control	DOA; Hereditary spastic paraplegia type 7	Charif et al. [168]
*AFG3L2*	604581	18p11.21	Mitochondrial quality control; mitochondrial protein synthesis, and cellular respiration	DOA; spinocerebellar ataxia type 28; spastic ataxia type 5	Ghosh et al. [169]
*DNM1L*	603850	12p11.21	Mitochondrial fission	DOA; myoclonic epilepsy	Sylvie et al. [170]; De Souza Crippa et al. [171]
*SSBP1*	600439	7q34	mtDNA replication	DOA; cardiomyopathy; retinal dystrophy; retinopathy; deafness	Del Dotto et al. [172]
*WFS1*	606201	4p16.1	Calcium homeostasis in endoplasmic reticulum	Wolfram syndrome (hear impairment, diabetes mellitus, and optic atrophy)	de Mujinck et al. [173]
*NR2F1*	132890	5q15	Transcriptional regulation	Bosch-Boonstra-Schaaf optic atrophy syndrome	Sara et al. [174]

*OMIM* online mendelian inheritance in man, *DOA* dominant optic atrophy, *MFN2* mitofusin 2 protein, *AFG3L2* AFG3 like matrix AAA peptidase subunit 2, *DNM1* dynamin 1 like, *SSBP1* stranded DNA-binding protein 1, *NR2F1* nuclear receptor subfamily 2, group F, member 1

LHON and DOA share some common characteristics: both exhibit a similar pattern of optic nerve axonal degeneration and chronic increase in ROS production. Due to the phenotypic similarity, the majority of therapies investigated for DOA are adapted from LHON protocols. These therapeutic approaches include antioxidants (vitamins B2, B12, C, E, folic acid and lipoic acid) [176], Idebenone (which showed improved visual functions in 5 of 7 DOA patients carrying *OPA1* haploinsufficient mutations in an open-label trial) [152], and mitochondrial modulators (Zolpidem, Papaverine, and QS10) [177]. Gene therapy using CRISPR-Cas9 and AAV-mediated wild-type *OPA1* expression is in preclinical development [178], with CRISPR-based gene editing proven successful in vitro for the *OPA1* missense mutation [179]. Animal models carrying *OPA1* truncated mutations, which share a 96% identical amino acid sequence, are available for efficacy and safety assessment [180]. A recent study revealed that abnormal increase in autophagic activity at the axonal hillock leads to the depletion of mitochondria in RGC axons, resulting in a dying-back type of axonopathy that was reversed when active AMPK was inhibited [181].

#### Other mitochondrial optic neuropathies

Chronic progressive external ophthalmoplegia (CPEO) is characterized by gradual onset of bilateral ptosis and symmetric ophthalmoplegia caused by genetic mutations impairing oxidative phosphorylation, with an estimated prevalence of 3.39 per 100,000 in UK. CPEO displays notable genetic heterogeneity, being sporadic in around 50% of cases involving single large de novo mtDNA deletion [182], with inherited forms involving mutations in genes such as *RRM2B, TWNK, OPA1, POLG*, and *ANT1* [183]. Treatment is primarily surgical for visually obstructive ptosis, supplemented by prismatic glasses for strabismus [184].

Kearns-Sayre Syndrome (KSS) is caused by large-scale mtDNA deletion (1.1 to 10 kb), presenting before age 20 with retinal pigmentary degeneration, CPEO, and cardiac conduction block [154, 185]. The most frequently identified deletion in KSS is a 4,977 bp deletion (NC_012920.1:M.8483_13459del) [186]. Treatment options are limited, though patient-specific iPSC-based therapies and mitochondria augmentation therapy (MAT) show promise. Jacoby et al. treated 6 patients with single large-scale mtDNA deletion syndromes using hematopoietic cells enriched with exogenous mitochondria, demonstrating clinical improvements [187].

Wolfram syndrome-1 (WS1) is primarily caused by mutations in the *WFS1* gene encoding the ER-resident wolframin protein, presenting with diabetes mellitus, optic atrophy, neurological deficits, and hearing impairment [173]. Increasing evidence reports dysfunctional ER-mitochondria communication caused by wolframin deficiency, with impaired complex I- and complex II-driven respiration [188, 189]. Treatment options include GLP-1 analogs for diabetes, with clinical trials evaluating sodium valproate (NCT03717909) and advanced modalities including gene therapy and stem cell approaches [190–192]. Details about the drug treatment options that have been investigated in patients with WS1 can be found in Table 4.

**Table 4:** Drugs that have been investigated in patient with WS1 syndrome

*Compounds*	*Mechanisms of action*	*Status of use*	*References*
Dantrolene sodium	A hydantoin derivative skeletal muscle relaxant; inhibits ER calcium efflux through ryanodine receptors	Clinical trial in adult and pediatric WS patients	Abreu et al. [193]; Toppings et al. [194]
Carbachol	A muscarinic agonist which enhances the secretion of insulin stimulated by glucose and mobilizes intracellular calcium stores	The experimental study showed the administration of carbachol can enhance insulin secretion	Tools et al. [195]
Rapamycin	Suppresses IP3 receptor and enhances SERCA activation	The experimental study demonstrated that rapamycin could prevent ER-mediated β-cell death and calcium efflux from the ER	Hara et al. [196]
Pioglitazone	Reduces IP3 receptor mediated calcium release from ER	Has been tested in adults diagnosed with WS1 diabetes	Akiyama et al. [197]; Sobhani et al. [198]; Hara et al. [196]
Valproate acid	A histone deacetylation enzyme inhibitor that enhances the acetylation level of histone and improves gene transcription	Clinical trials in patients with WS1	Batjargal et al. [199]; Sun et al. [200]
Liraglutide, dulaglutide, semaglutide, exenatide	GLP-1 receptor agonists that interfere with the unfolded protein response in ER	Clinical trials in children and adolescents with type 2 diabetes	Tamborlane et al. [201]; Toomas et al. [202]; Frontino et al. [203]
Dipeptidyl peptidase-4	Enhances GPL-1 concentration; ameliorates pancreatic β-cell failure	Has been tested in the mouse model of WS and in patients diagnosed with WS1	Tarcin et al. [204]

*WS1* Wolfram syndrome 1, *ER* endoplasmic reticulum, *IP3* inositol triphosphate, *SERCA* sarcoendoplasmic reticulum Ca^2+^-ATPase, *GPL-1* glucagon-like peptide-1

#### Mitochondrial dysfunction in glaucoma

Glaucoma is a neurodegenerative ocular disease characterized by progressive loss and dysfunction of RGCs. Primary open-angle glaucoma (POAG) is the most prevalent subtype. Current therapeutic strategies primarily target intraocular pressure (IOP) management through eyedrops, lasers, implantable devices, and surgery. However, many patients still experience vision loss even when their IOP is within the physiological range, highlighting the need for IOP-independent neuroprotective strategies [205]. Unlike primary mitochondrial optic neuropathies, glaucoma involves elevated IOP, vascular dysregulation, and neuroinflammation converging upon mitochondrial dysfunction as a critical downstream pathway.

Direct bioenergetic deficits including impaired complex I function in lymphoblasts and peripheral blood mononuclear cells [206, 207], pathogenic mtDNA variants in 50% patients of a POAG cohort [208], and increased ROS with reduced ATP levels in trabecular meshwork cells have been documented [209]. Experimental models recapitulate these findings: RNA-sequencing in DBA/2 J mice revealed significant enrichment of differentially expressed genes in the mitochondrial dysfunction and OXPHOS pathways [210]. Loss of synapse and reduction in the volume of mitochondria cristae have also been found in the dendrites of RGCs, promoting excessive fission and impairing ATP production [211]. Genetic studies further implicate mitochondrial defects across glaucoma subtypes, with the common 4,977 bp mtDNA deletion significantly increased in POAG trabecular meshwork [212], and variants in complex I and complex III associated with disease risk.

Age-dependent decline in NAD^+^ levels in the retina makes retinal neurons vulnerable to IOP-related stress, and low serum nicotinamide levels have been found in glaucoma patients [213]. Upregulation of NAD^+^ level via oral nicotinamide has shown neuroprotective effects on RGC axons, somas, and dendrites in vivo, increasing mitochondrial motility and size while improving OXPHOS [210]. AAV-mediated overexpression of NMNAT2, a terminal enzyme for NAD^+^ production, provided robust neuroprotection in experimental glaucoma models [214]. Based on these findings, there has been a surge in the development of potential neuroprotective therapies targeting mitochondrial metabolism (Table 5). One completed clinical trial found that oral nicotinamide treatment for six months improved visual functions in patients with glaucoma according to electroretinography and perimetry measurements [232]. Currently, several clinical trials (including NCT0397469) are ongoing.

**Table 5:** Potential neuroprotective treatments focus on mitochondria in experimental models of glaucoma

*Treatments*	*Models*	*Targets/Functions*	*References*
Nicotinamide	DBA/2 J mouse model; rat ocular hypertensive model; mouse retinal axotomy model	Precursor of NAD	Williams et al. [210, 215]; Tribble et al. [216]
Coenzyme Q10	Retinal ischemia reperfusion mouse/rat model; hypertonic saline episcleral vein injection rat model; DBA/2 J mouse model	Cofactor of the electron transport chain	Ju et al. [217]; Nucci et al. [218]; Lee et al. [219]
Citicoline	Acute IOP elevation rat model; cross-linking hydrogel induced chronic IOP elevation rat model; mouse retinal explants; kainic acid-induced retinal degeneration; optic nerve crush rat model	Precursor of phosphatidylcholine and acetylcholine	Merwe et al. [220]; Oshitari et al. [221]; Parisi et al. [222]; Park et al. [223]
Resveratrol	Retinal ischemia reperfusion rat model; serum deprivation R28 cell model; optic nerve crush mouse model; human glaucomatous trabecular meshwork cell model; steroid-induced ocular hypertension rat model	Multiple targeted pathway	Vin et al. [224]; Pang et al. [225]; Lindsey et al. [226]; Avotri et al. [227]
Pyruvate	DBA/2 J mouse model; magnetic bead mouse model of ocular hypertension	Diverging energy metabolism pathways (such as gluconeogenesis and glycolysis)	Harder et al. [228]; Mohammad et al. [229]
Ketone-based treatments	DBA/2 J mouse model; optic nerve crush mouse model; rat model of NMDA-induced damage of RGC	Promoting mitochondrial biogenesis	Mohammad et al. [230]; Thaler et al. [231]

*NAD* nicotinamide adenine dinucleotide, *IOP* intraocular pressure, *NMDA* N-methyl-D-aspartate, *RGC* retinal ganglion cell

Targeting mitophagy has also emerged as a promising therapeutic strategy, with AAV2-mediated Parkin overexpression significantly attenuated RGC death in chronic high IOP rat models [233]. Human genetics further validates this axis, as mutations in the mitophagy receptor OPTN and its activating kinase TBK1 are established risk factors for POAG [234]. Functional studies demonstrate that pathogenic E50K mutation in OPTN increases RGC susceptibility to cell death, which can be rescued by the TBK1 inhibitor Amlexanox [235]. CoQ10 treatment has shown preclinical efficacy in preventing RGC loss and preserving mtDNA content in glaucomatous DBA/2 J mice, though its lipophilic nature limits bioavailability in humans [219]. These findings underscore mitochondrial health as a central therapeutic target in glaucoma, with multiple clinical trials underway to evaluate the efficacy of neuroprotective strategies targeting mitochondrial metabolism.

## Therapeutic landscape: current advances and translational challenges

The past decade has witnessed landmark therapeutic milestones in mitochondrial medicine: Idebenone received European approval for LHON, omaveloxolone became the first FDA-approved therapy for Friedreich’s ataxia in 2023, and tofersen achieved regulatory approval for SOD1-associated ALS the same year. Across the disease sections discussed above, a rich pipeline of gene therapies, mitophagy enhancers, metabolic modulators, and organelle replacement strategies are actively advancing through preclinical and clinical evaluation. Nevertheless, current therapeutic strategies face significant and interconnected biological, technical, and clinical challenges. A critical appraisal of these limitations, many of which are sharply delineated in the context of optic neuropathies, reveals key translational gaps that are broadly relevant to mitochondrial medicine in neurodegeneration.

First, viral vector-based gene therapies, while promising for monogenic disorders like LHON, could disturb the immune system with unpredictable biodistribution (such as contralateral effects observed after unilateral intravitreal injection), and dose-limiting inflammatory responses [236]. Similarly, stem cell-based regenerative strategies face unresolved issues of poor long-term graft survival, inadequate synaptic integration into host retinal neuronal circuitry, and inherent tumorigenicity risks, particularly with iPSC-derived lineages. These technical hurdles underscore a universal challenge in neurodegenerative therapy to achieve a safe, sustained, and anatomically precise delivery of therapeutic payloads [237].

Second, the efficacy of pharmacological agents is constrained by fundamental delivery and engagement barriers. Small molecules such as Idebenone often exhibit poor bioavailability, variable mitochondrial uptake across tissues, and limited penetration of critical barriers, notably the blood-retinal and blood–brain barriers [238]. Furthermore, low dose and long-term drug administration raises concerns about mitochondrial hormesis, where prolonged metabolic modulation may paradoxically exacerbate dysfunction, and off-target effects that can disrupt essential redox signaling pathways. The failure of several large-scale antioxidant trials, including high-dose coenzyme Q10 in Parkinson’s disease, highlights that simply scavenging ROS without addressing upstream mitochondrial defects may be insufficient.

Third, the variable disease progression observed even within monogenic disorders highlights a critical need for robust stratification biomarkers, such as metabolomic signatures, mitochondrial DNA copy number, circulating cell-free mtDNA levels, and quantitative imaging metrics (OCT for optic neuropathies, FDG-PET for AD), to identify patients within optimal therapeutic windows. This heterogeneity complicates clinical trial design and interpretation. Moreover, multiple preclinical animal models fail to recapitulate the slow, episodic neurodegeneration characteristic of human diseases, leading to a systematic overestimation of therapeutic efficacy that hampers translational prediction [239]. For instance, acute toxin-based Parkinson’s disease models (MPTP, 6-OHDA) produce rapid neurodegeneration that does not reflect the decades-long prodromal phase of human Parkinson’s disease.

Fourth, a pivotal insight from mitochondrial research is that bioenergetic rescue does not guarantee neuronal survival. Partial restoration of ATP levels in experimental models may not linearly correlate with long-term neuronal preservation, suggesting that metabolic support alone may be insufficient to counteract entrenched secondary degenerative pathways, such as sustained neuroinflammation or apoptotic commitment [240]. This principle implies that for many neurodegenerative diseases, combination therapies targeting both primary mitochondrial dysfunction and downstream consequences will likely be necessary.

Finally, for prevalent neurodegenerative diseases discussed in this review, the multifactorial nature of mitochondrial involvement presents an additional layer of complexity. Unlike monogenic mitochondrial disorders where a single genetic target can be addressed, diseases such as AD and Parkinson’s disease involve multiple converging pathways of mitochondrial damage, necessitating multimodal therapeutic approaches and careful patient stratification based on the predominant mitochondrial deficit. The emerging concept of “mitochondrial medicine” must therefore evolve from single-target interventions toward systems-level approaches that consider the interplay between bioenergetics, dynamics, quality control, and the broader cellular ecosystem including glial support networks.

## Conclusions and future perspectives

Mitochondrial dysfunction has emerged as a unifying pathological hub across the spectrum of neurodegenerative diseases. From the well-characterized monogenic optic neuropathies and Friedreich’s ataxia to the complex, multifactorial pathology of Alzheimer’s and Parkinson’s diseases, the shared themes of bioenergetic failure, disrupted dynamics, defective quality control, and neuroinflammatory amplification underscore the central role of mitochondrial health in neuronal survival. Importantly, the approval of omaveloxolone for Friedreich’s ataxia and Idebenone for LHON demonstrates that mitochondria-targeted therapies can achieve regulatory milestones, providing a framework for future drug development.

Studies of inherited optic neuropathies have highlighted that discrete genetic lesions in mtDNA or nDNA converge on mitochondrial dysfunction, ultimately triggering the demise of selectively vulnerable neurons. The persistent enigmas of these disorders, including incomplete penetrance, delayed onset related to age, and sex bias, highlighting the complex crosstalk between genetic susceptibility and other modifying factors, offering a new perspective to investigate the broader dynamics of neurodegenerative processes.

A better understanding of the molecular basis of mitochondrial dysfunction is needed. Cellular models, including cybrids (a hybrid cell with nuclear genes from one cell and mitochondrial genes from another cell), as well as differentiated cybrids with neuronal like properties, can complement genetic analysis like linkage analysis of extensive pedigrees and deep sequencing of the mitochondrial genome. Moreover, the establishment of reliable animal models that faithfully mimics optic nerve degeneration observed in humans, such as patient-derived retinal organoids and non-human primate models of optic neuropathy, would be a valuable resource for testing innovative and promising therapeutic interventions. These advanced models should be coupled with cutting-edge imaging modalities, such as fluorescence lifetime imaging microscopy, to enable real-time monitoring of mitochondrial functions and therapeutic response in vivo.

Overcoming biological barriers is another universal challenge. For the eye and the brain alike, the development of next-generation delivery platforms, such as engineered AAV capsids with enhanced retinal tropism and mitochondrially targeted nanoparticles, can help improve therapeutic biodistribution while minimizing systemic exposure.

Additionally, a more comprehensive and systematic investigation of the progression of these diseases is needed. Long-term follow-up incorporating neuroimaging, functional assessments, and proteomic analysis may help define the most relevant criteria and endpoints for future clinical trials design. The implementation of multi-omics approaches will benefit patient stratification and trial design. Comprehensive profiling of mitochondrial DNA heteroplasmy, metabolic signatures, and nuclear modifier genes in well-characterized patient cohorts may identify predictive biomarkers of disease progression and treatment responsiveness.

Furthermore, establishing international collaborative networks will be helpful to standardize research protocols and accelerate therapeutics development. Such consortia could facilitate the creation of shared biorepositories, harmonize outcome measures across clinical studies, and promote knowledge exchange between basic scientists and clinicians. This interconnected system is indispensable for moving from a one-gene-one-disease perspective to a network-based understanding of pathology, which is also applicable to complex disorders like Parkinson’s and Alzheimer’s disease.

In conclusion, the strategies outlined, including advanced modeling, precision delivery, biomarker-driven patient selection, and collaborative systems medicine, constitute a convergent roadmap. By integrating insights from both tractable visual system models and complex systemic neurodegenerative diseases, we can generate principles, tools, and therapeutic paradigms that will accelerate the development of effective, mitochondria-targeted interventions for the vast spectrum of neurodegenerative disorders that share this common pathological hub. The convergence of advanced gene editing technologies, precision metabolomics, single-cell multi-omics, and artificial intelligence-driven drug discovery holds particular promise for transforming our understanding of mitochondrial neurodegeneration into clinically meaningful therapeutic advances in the coming decade.

## Data Availability

No datasets were generated or analyzed during the current study.
